# Recombinant thermotolerant alkaline lipase from *Lysinibacillus fusiformis* for detergent and hard (Ras) cheese applications: cloning, expression, molecular docking, and characterization

**DOI:** 10.1186/s12934-026-03084-w

**Published:** 2026-08-29

**Authors:** Ghada M. El-Sayed, Hala R. Wehaidy, Adel M. M. Kholif, Walaa H. Salama, Rasha G. Salim

**Affiliations:** 1 Microbial Genetic Department, National Research Centre, Dokki, Giza 12622 Egypt; 2 Chemistry of Natural and Microbial Products Department, National Research Centre, Dokki, Giza 12622 Egypt; 3 Dairy Sciences Department, National Research Centre, Dokki, Giza 12622 Egypt; 4 Molecular Biology Department, National Research Centre, Dokki, Giza 12622 Egypt

**Keywords:** *Lycinibacillus fusiformis*, Recombinant lipase A, Gene cloning, Heterologous expression, Enzyme characterization, Ras cheese ripening

## Abstract

**Background:**

Thermostable and alkaline lipases are of significant interest for industrial applications, particularly in detergents and food processing. This study aimed to isolate, clone, and express lipase-encoding genes from a potent bacterial source to produce a thermo-tolerant alkaline lipase with enhanced catalytic efficiency and practical applicability.

**Results:**

Among several bacterial isolates, the most potent lipase producer was identified as *Lysinibacillus fusiformis*, and its 16 S rRNA sequence was deposited in GenBank (PP757498). Three lipase-encoding genes (*est*, *est2*, and *lipA*) were successfully isolated, cloned, and heterologously expressed in *Escherichia coli* BL21 (DE3). Their sequences were submitted to GenBank under accession numbers PX136937.1, PX136938.1, and PX136936.1, respectively. The recombinant lipase encoded by *lipA* (rLipase) exhibited the highest activity (150 U/mL) compared with the native enzyme (56.2 U/mL). Molecular docking analysis demonstrated strong binding affinity of rLipase toward major fatty acid derivatives in olive oil, with the highest affinity for linoleic acid (− 8.0 kcal/mol), followed by oleic acid (− 7.8 kcal/mol) and palmitic acid (− 7.3 kcal/mol). These interactions were stabilized by hydrophobic interactions and hydrogen bonding, with key contributions from critical amino acid residues, particularly VAL250. The partially purified recombinant lipase (rLipase) exhibited a maximum activity of 320 U/mL at 80 °C and pH 9, demonstrating remarkable thermostability and alkaline tolerance. Functional evaluation showed that rLipase improved the detergent efficiency for oil stain-removal from cotton fabrics. In addition, supplementation with 0.4% rLipase accelerated Ras cheese ripening by shortening the maturation period from 120 to 90 days with maintaining the desired ripening process.

**Conclusions:**

The recombinant lipase from *Lysinibacillus fusiformis* demonstrated high thermal stability, alkaline tolerance, and strong catalytic efficiency. Its effectiveness in detergent formulations and cheese ripening highlights its potential as a versatile industrial biocatalyst for lipid bioconversion and related applications.

**Supplementary Information:**

The online version contains supplementary material available at https://doi.org/10.1186/s12934-026-03084-w.

## Background

Lipase (triacylglycerol acylhydrolase, EC 3.1.1.3) is a serine hydrolase that catalyzes the hydrolysis of mono-, di- and triglycerides into glycerol and fatty acids in an aqueous solution [1]. Bacterial lipases have considerable importance as they exhibit substantial diversity in molecular weight, amino acid composition, stability, substrate specificity, and ability to work in extreme conditions [2–4]. In order to be used in industry, lipases need to be thermostable and maintain their activity in organic solvents [5, 6]. Thermostability is required since many processes use temperatures around 50 °C due to the high melting points of the lipids that are used as substrate [7].

Lipases have broad industrial applications in the food, pharmaceutical, paper, cosmetic, detergent, wastewater treatment, and leather industries [8–10]. Among these, the detergent industry is the largest consumer, as lipases enhance the removal of lipid-based stains while enabling more environmentally sustainable detergent formulations. For detergent applications, lipases must retain high catalytic activity and stability under alkaline conditions and in the presence of surfactants, oxidizing agents, and other formulation components [11]. In the dairy industry, lipases are widely employed to hydrolyze milk fat, modify the fatty acid chain lengths, enhance cheese flavor, and accelerate cheese ripening through controlled lipolysis of milk fat, butter, and cream [12].

Bioinformatics, together with advances in gene cloning and recombinant DNA technology, has become a powerful tool for exploring protein structure–function relationships, predicting enzyme properties, and identifying promising microbial candidates for enhanced lipase production [13, 14]. These integrated approaches have significantly accelerated enzyme engineering and heterologous expression strategies, enabling improved lipase yields and the tailoring of enzyme properties for specific industrial applications [15]. Despite these advances, large-scale production of bacterial lipases remains challenging. Native production levels are often low, and conventional methods—such as strain improvement and fermentation optimization—have achieved only limited success in meeting industrial demands. Although genetically engineered strains and optimized expression systems have been developed to address these limitations [13], progress is still constrained by an incomplete understanding of the molecular mechanisms regulating lipase gene expression. Therefore, further investigation of these regulatory pathways is essential to enhance lipase production and broaden its industrial and biotechnological applications.

Computational approaches leverage a wide range of structural and physicochemical features of protein sequences to enable comprehensive enzyme analysis and characterization. These methods provide valuable insights into the relationships between protein structure, function, and substrate interactions [16]. To address the complexity of protein architecture, the template-based homology modeling is widely regarded as one of the most reliable and accurate techniques for predicting three-dimensional structures. By applying these approaches, protein models can be constructed and evaluated to identify key structural features that influence lipase function, substrate specificity, and catalytic behavior [17]. This structural understanding has facilitated the development of semi-rational protein engineering, an effective strategy for redesigning lipases to enhance catalytic efficiency, improve substrate binding, and optimize overall enzyme performance. Collectively, these advances have significantly broadened the industrial, agricultural, and biotechnological applications of lipases.

The present study was conducted as an attempt for the production of a recombinant thermostable alkaline lipase from *Lycinibacillus fusiformis*. We have successfully cloned and expressed lipase-encoding genes, resulting in enhanced lipase production. Furthermore, our findings highlight the value of in silico analysis of the lipase encoded by the **LipA** gene, which provided improved insights into enzyme–substrate binding and catalytic activity predictions. The study also **has** investigated the potential of the recombinant lipase enzyme in some industries, including **the** detergent and cheese industries.

## Methods

### Isolation of microorganisms and plate screening assays for lipolytic activity

Microorganisms were isolated from freshly collected milk samples obtained from farms in the Giza Governorate. Samples were collected in sterile plastic containers and transported to the laboratory under aseptic conditions to ensure the integrity of potential lipase-producing bacteria.

For isolation, 1 mL of each sample was mixed with 9 mL of sterile distilled water and subjected to heat treatment at 80 °C for 10 min to eliminate most vegetative cells. The treated samples were then serially diluted (10⁻⁵ to 10⁻⁹) and plated on tributyrin agar (TBA) at pH 7. Plates were incubated at 37 °C for three days. Colonies exhibiting clear zones, indicative of lipolytic activity, were selected, purified, and subcultured on TBA under the same conditions to confirm zone formation [18, 19]. The isolate showing the largest clear zone was selected for further investigation and designated **NRC420**.

### Quantitative assessment of lipase-producing isolates

#### Lipase production by the most potent bacterial isolate

The most active lipase-producing isolate was selected for quantitative evaluation of enzyme production. Here, two production media were used: the first consisted of tryptone soya broth supplemented with 1% tributyrin. The second consisted of (g/L): glucose (25.0), yeast extract (15.0), tryptone (15.0), sodium acetate (10.0), MgSO_4_.7H_2_O (0.2), KCl (0.5), and olive oil (1% v/v), adjusted to pH 8.0. Cultures were incubated at 40 °C with shaking at 200 rpm for 72 h [20]. The inoculum was prepared by introducing a loopful of bacterial culture into 50 mL of the production medium, followed by incubation at 40 °C and 200 rpm for 24 h. Subsequently, 2.5 mL of the prepared inoculum was transferred into 50 mL of fresh production medium and incubated under the same conditions for 72 h. After incubation, the culture was centrifuged at 4000 rpm, and the supernatant was collected as the crude lipase enzyme extract.

#### Lipase assay

Lipase activity was determined according to Ertuğrul et al. [21] using *p*-nitrophenyl palmitate (pNPP) (Sigma, USA) as the substrate. The substrate solution was freshly prepared by combining solution A and solution B. Solution A was prepared by dissolving 30 mg of pNPP in 10 mL of isopropanol. Solution B consisted of 0.1 g gum Arabic and 0.4 mL Triton X-100 dissolved in 90 mL of 50 mM Tris–HCl buffer (pH 8), with continuous stirring until completely solubilized. For the assay, 9 mL of the substrate solution was mixed with 1 mL of the enzyme solution and incubated at 60 °C for 30 min. Lipase activity was then determined by measuring the absorbance at 410 nm.

### Molecular identification of the most potent isolate

#### Genomic DNA purification and PCR amplification of the 16 S rRNA gene

Genomic DNA was extracted from **the** lipase-producing isolates using the Thermo Scientific GeneJET Kit following the manufacturer’s protocol. The 16 S rRNA gene was amplified by PCR using universal primers 8 F and 1492R [22] with Emerald Amp GT PCR master mix. PCR conditions included initial denaturation at 94 °C for 3 min, followed by 35 cycles of 94 °C for 30 s, 50 °C for 30 s, and 72 °C for 45 s, with a final extension at 72 °C for 10 min. The amplified products were analyzed on a 1% agarose gel, stained with ethidium bromide, and visualized under UV light. Sequencing of the 16 S rRNA gene was performed using the Sanger method [23] with a BigDye X Terminator kit on a 3500 genetic analyzer. The obtained sequences were analyzed using the NCBI BLASTN tool and compared with database sequences.

#### Phylogenetic analysis

The obtained sequence was edited using BioEdit 7.1.10 software [24]. To identify similar species, the edited sequence was compared to those in the GenBank database (http://www.ncbi.nlm.nih.gov/blast). A total of 12 related species’ 16 S rRNA gene sequences from GenBank were used for phylogenetic tree construction. Multiple sequence alignment was performed using the MUSCLE algorithm [25] within MEGA11 [26]. The evolutionary history was inferred using the neighbor-joining method [27] with 1000 bootstrap replications [28], and evolutionary distances were calculated using the Jukes–Cantor method [29].

### Molecular cloning and heterologous expression of lipase encoding genes

#### Bacterial strains, growth media, and plasmids

The bacterial strains employed in this investigation included bacterial isolate **NRC420**, which served as the source for isolation of lipase encoding genes. *Escherichia coli* DH5α was used as the host strain for cloning vectors construction, whereas *Escherichia coli* BL21 (DE3) functioned as the expression host for recombinant lipase production. Both *E. coli* strains were purchased from Invitrogen (San Diego, CA, USA). All strains were grown and preserved in Lysogeny Broth (LB) medium [30] for purposes including DNA extraction, cloning procedures, and plasmid amplification. The lipase encoding genes were initially cloned using the CloneJET PCR Cloning Kit (Thermo Scientific, USA) and subsequently propagated in *E. coli* DH5α. For recombinant protein expression, the pET-28a (+) plasmid (Novagen, CA, USA) was employed as the expression vector in *E. coli* BL21 (DE3).

#### Primer design and PCR amplification of lipase encoding genes

In this step, the complete genome sequence of *Lysinibacillus fusiformis* strain RB-21 (NCBI Reference Sequence: NZ_CP010820.1) was retrieved from the NCBI database to identify lipase-coding genes. Candidate genes were screened based on sequence homology and conserved domain analysis using the NCBI Conserved Domain Database (CDD; https://www.ncbi.nlm.nih.gov/Structure/cdd/wrpsb.cgi).

Three lipase-encoding genes were selected due to the presence of conserved lipase domains and their predicted efficiency in olive oil degradation. The selected genes correspond to the following Gene IDs: 29443290, 29439326, and 29440449. Three primer pairs were designed, one for each selected gene. Primer design was performed using Primer3Plus (https://www.primer3plus.com). Restriction enzyme recognition sites were selected using NEBcutter (https://nc3.neb.com/NEBcutter/) and incorporated at the 5′ ends of both forward and reverse primers to facilitate subsequent cloning. The complete primer sequences, including the added restriction sites (underlined), are listed in Table 1. Extracted genomic DNA was used as a template for amplification of the lipase-encoding genes. The efficiency and specificity of the synthetically designed primers were first evaluated by PCR amplification. The PCR program consisted of an initial denaturation step at 94 °C for 3 min, followed by 35 cycles of denaturation at 94 °C, annealing at the temperatures listed in Table 1, and extension at 72 °C for 2 min. A final extension step was performed at 72 °C for 10 min. The gene fragments intended for cloning were subsequently amplified using Phusion High-Fidelity DNA Polymerase (Thermo Fisher Scientific, USA) according to the manufacturer’s instructions. For this amplification, the annealing temperature was set at 62 °C.

**Table 1:** Primer sequences used for PCR amplification of lipase-encoding gene fragments

Gene ID	Primer sequence Sequence (5’->3’)	Restriction enzymes	Annealing temperature	Expected product length (bp)
29,443,290	*GGATCC*ATGTATTACAGCAATGGTAACTATG	BamH1	45 °C	1773
	*GAGCTC* CTTTCAGTAAGTCATAAATAATCG	Sac1		
29,439,326	*GGATCC*ATGGAAGGTGGTCAAATGGAC	BamH1	55 °C	945
	*AAGCTT*TCCTCGAAGCCTATTGCCTTG	HindIII		
29,440,449	*GGATCC*ATAGGTGATAGCTTAACAGAAGG	BamH1	50 °C	702
	*GAGCTC* TTAGAACCTCCCGATTTTGTCTC	Sac1		

#### Cloning of lipase encoding genes and sequence analysis

The amplified PCR product was separated on a 1.2% agarose gel, and the DNA bands corresponding to the target genes were excised and purified using the GeneJET Gel Extraction Kit (Thermo Scientific). The purified lip genes fragments were then ligated into the pJET1.2/blunt cloning vector using the CloneJET PCR Cloning Kit (Thermo Scientific) according to the manufacturer’s guidelines. Transformation of ligation reactions was implemented into Competent *E. coli* DH5α cells which then was permitted to grow on LB agar plates supplemented with carbenicillin (50 µg/mL) and incubated at 37 °C for 18 h. Positive true transformants were identified by colony formation. The resulting recombinant construct was designated by gene symbol-pJET.

#### Heterologous expression of lipases in *E. coli* BL21 (DE3) and gene sequencing

The recombinant plasmids were extracted from confirmed transformants using the GeneJET Plasmid Miniprep Kit (Thermo Scientific) according to the manufacturer’s protocol. Both recombinant constructs and the pET-28a (+) expression vector were subjected to double digestion using the restriction enzymes specified in Table 1 to facilitate subcloning of the target gene. Following ligation of the insert into pET-28a (+), the recombinant plasmids were transformed into competent *E. coli* DH5α cells and plated on LB agar supplemented with kanamycin (50 µg/mL) for selection. All cloning and transformation procedures were carried out in accordance with the methods described by Sambrook et al. [31]. Colonies that grew under kanamycin selection were picked and cultured in LB broth containing 50 µg/mL kanamycin for plasmid propagation. Positive aprE-pET28a recombinants were verified by double digestion of the recombinant plasmid with the corresponding restriction enzymes. After confirming the correct construct and plasmid integrity, the verified recombinant vector was isolated and transformed into competent *E. coli* BL21 (DE3) cells for protein expression under kanamycin selection. The latest construct was designed gene symbol- pET. The purified PCR amplicon was sequenced by sequencer 3500 genetic analyzer, big dye X terminator kit (Termo Fisher, USA) in biomedical laboratory of colors (Clinilab, Egypt). using pET28a(+) vector-specific primers: pET-F (5′-CGTCCGGCGTAGAGGATC-3′) and pET-R (5′-ATCCGGATATAGTTCCTCCTTTC-3′). The resulting nucleotide sequence was edited by trimming both the 3′ and 5′ terminal regions using BioEdit version 7.2 [24]. The refined sequence was then compared with entries in the NCBI nucleotide database using the BLAST tool and subsequently submitted to obtain an accession number.

To validate the sequence and structural alignment and to elucidate the evolutionary relationships and sequence conservation among oleate hydratases, representative homologous protein sequences were retrieved from the GenBank database using Position-Specific Iterated BLAST (PSI-BLAST). Structure-based multiple sequence alignment, including the superimposed secondary structure elements, was performed using ESPript 3.0 [32]. Conserved domains in the deduced amino acid sequence were identified using the NCBI Conserved Domain Database (CDD) (https://www.ncbi.nlm.nih.gov/Structure/cdd/wrpsb.cgi).

### Protein expression, partial purification and SDS-PAGE

After confirming successful transformation and correct construction of the Lip-pET recombinant plasmid, verified clones were grown in LB medium containing kanamycin at 37 °C until the culture reached mid-log phase (OD₆₀₀ ≈ 0.4). Protein expression was then induced by adding isopropyl β-D-1-thiogalactopyranoside (IPTG) at final concentrations of 0.1, 0.2, 0.3, 0.4, 0.5, and 1.0 mM. Induction was carried out at different temperatures (18, 28, and 37 °C) to optimize expression conditions. Following induction, the cells were harvested by centrifugation, washed with deionized water, and resuspended in 10 mmol/L phosphate-buffered saline (PBS, pH 7.4). Cell disruption was performed by sonication for a total of 10 min using 30-second pulses alternated with 30-second cooling intervals on ice. The lysate was then centrifuged to remove cell debris. Proteins in the supernatant were precipitated by absolute Ethyl alcohol solution with different concentrations (25–75%). Then the produced fractions were dried and assayed for lipase activity. The fraction with the highest purification fold was used for lipase characterization and application. The total protein concentration was determined using the Lowry assay [33]. Protein samples (10 µg) were mixed with Laemmli loading dye and separated by SDS–PAGE under the conditions described by Laemmli [34]. Protein bands were visualized using Coomassie Brilliant Blue staining. A PageRuler™ Unstained Broad Range Protein Ladder was used as the molecular weight marker. For silver staining, the gel was processed according to the protocol described by Shevchenko [35].

### Computational study

#### Template search and homology modeling

The three-dimensional structure of the selected protein was constructed using a homology modeling approach. Potential structural templates were identified by performing a PSI-BLAST search against the Protein Data Bank (PDB) [36]. Only templates showing greater than 30% sequence identity were selected for modeling target protein. Structural models were generated with MODELLER version 10.6 [37] using sequence alignments between the target protein and experimentally resolved structures. The resulting models were assessed based on TM-score, root mean square deviation (RMSD), and the discrete optimized protein energy (DOPE) score. Additional structural validation was conducted using online tools, including VERIFY3D [38] and PROCHECK [39] to evaluate the stereochemical quality and overall reliability of the predicted models.

#### Molecular docking analysis

Molecular docking analysis was conducted to explore the interactions between the predicted lipase structure and three major fatty acids commonly found in olive oil: oleic acid (PubChem CID: 445639), linoleic acid (PubChem CID: 5280450), and palmitic acid (PubChem CID: 985). The putative active-site residues of the target enzyme were identified using the COACH server [40], which combines TM-SITE and S-SITE algorithms to predict potential ligand-binding regions. Prior to docking, hydrogen atoms were added to the lipase three-dimensional structure using AutoDock Vina’s MGL Tools [41]. Ligand structures were initially converted to mol2 format using Open Babel [42] and subsequently transformed into pdbqt format with AutoDock Tools for docking preparation. Docking simulations generated ten binding conformations for each ligand, and the optimal pose was selected based on the lowest predicted binding energy. Visualization and analysis of the receptor–ligand interactions, including ligand orientation, hydrogen bonding, and key amino acid contacts within the binding pocket, were performed using BIOVIA Discovery Studio Visualizer (version 2021, Dassault Systèmes, San Diego, CA, USA). All generated docking poses were examined, and the conformation exhibiting the highest binding affinity was chosen for detailed evaluation. Two-dimensional interaction maps were created using the “Show 2D Diagram” tool, while hydrogen bond distances and other interaction parameters were assessed using the “Show Distance” function. All molecular docking calculations were carried out using AutoDock Vina 1.0 as described by Eberhardt et al. [41]

### Characterization of the partially purified recombinant *Lysinibacillus fusiformis* lipase (rLipase)

The biochemical properties of the partially purified recombinant *Lysinibacillus fusiformis* lipase (rLipase) were characterized by evaluating the effects of reaction temperature, pH, metal ions, surfactants, and commercial laundry detergents on enzyme activity. All experiments were performed in triplicate under the standard lipase assay conditions.

#### Effect of reaction temperature

The effect of temperature on the activity of the partially purified rLipase was studied by carrying out the reaction at different temperatures (from 50 to 90 °C), while keeping the other assaying conditions constant [43]. The temperature corresponding to the highest enzyme activity was considered as the optimum temperature. The activation energy (*Ea*) was calculated from Arrhenius plot:

$${\text{Slope }}= - {\mathrm{Ea}}/2.303{\text{ }}R,$$

Where R is the gas constant (*R* = 8.314 J/Kmol) [44].

#### Effect of reaction pH

The effect of reaction pH on the activity of the partially purified rLipase was studied by carrying out the reaction at different pH (from 7.5 to 10.5 using appropriate buffer systems) at the optimum assay temperature. The pH that yielded the highest lipase activity was considered as the optimum [45].

#### Effect of some metal ions on *Lycinibacillus fusiformis* rLipase activity

The effect of some metal on rLipase activity was investigated at the optimum reaction conditions. Calcium (Ca²⁺), magnesium (Mg²⁺), manganese (Mn²⁺), copper (Cu²⁺), and barium (Ba²⁺) ions were individually added to the reaction mixture at the desired concentration, whereas a reaction without added metal ions served as the control (100% activity). The residual enzyme activity was expressed as a percentage relative to the control [46].

#### Effect of some surfactants and commercial detergents on lipase activity

The effect of some surfactants including: Sodium dodecyl sulphate (SDS), Tween 20, Tween 80 and Triton X-100 on lipase enzyme activity was investigated at the optimum reaction conditions. They were individually supplemented with the reaction mixture at the desired concentration. The reaction mixture without surfactant was used as the control (100% activity), and the residual lipase activity was expressed relative to the control [46]. The compatibility of rLipase with some commercial detergents such as Ariel gel and powder, Persil gel, Oxi gel and Tide powder was also investigated. The detergent solutions were prepared at the desired concentration and incubated with the enzyme under the optimum assay conditions. Residual lipase activity was then determined and expressed as a percentage relative to the control without detergent [43].

### Evaluation of *Lysinibacillus fusiformis* rLipase in detergent formulation

The partially purified rLipase from *Lysinibacillus fusiformis* was evaluated as a detergent additive based on its ability to remove vegetable oil stains from white cotton fabric pieces (5 × 5 cm). Three experiments were carried out in 250 mL Erlenmeyer flasks as follows:

- 100 ml Tris-HCl buffer (pH 9.0) + 2 mL partially purified lipase (50 U/mL) + stained cloth.
- 50 mL of 1% Ariel detergent solution + 52 mL distilled water + stained cloth.
- 50 mL of 1% Ariel detergent solution + 50 mL Tris-HCl buffer (pH 9.0) + 2 mL partially purified lipase (50 U/mL) + stained cloth.

All flasks were incubated at 80 °C for 45 min with shaking at 50 rpm. After incubation, the cloth samples were rinsed twice with running water. Cleaning efficiency was calculated using the following equation [47]:

$$\begin{aligned}&\text{Cleaning efficiency} (\%)\\&\quad = [(\text{Weight before washing}\\ &\quad\quad-\text{Weight after washing})\\&\quad\quad /\text{weight before washing}]\\&\quad\quad\times 100.\end{aligned}$$

All experiments were performed in triplicate, and the values represent the mean of three replicates.

### Evaluation of *Lycinibacillus fusiformis* rLipase in Ras cheese manufacture

#### Milk and starter culture

Fresh cow and buffalo milk **were** obtained from the Faculty of Agriculture farm, Cairo University, Egypt. Microbial rennet powder (Chy-Max; Chr. Hansen Holding A/S, Bøge, 2970 Hørsholm, Denmark) was used for cheese production at a concentration of 1 g per 100 L of milk. Starter cultures of ***Lactococcus lactis*** subsp. *lactis* and ***Lactococcus lactis*** subsp. *cremoris* were obtained from the Food Science Department, Faculty of Agriculture, Ain Shams University, Egypt.

#### Cheese manufacture

Ras cheese manufacture was carried out as illustrated by Hofi et al. [48] and Hammam et al. [49]. The milk was heat-treated at 72 °C for ~ 15 s, then cooled to 37 °C. Ras cheese treatments were divided into three equal portions as follows: The first portion (control) was manufactured without adding **the** enzyme. The second (T1) and third portions (T2) were manufactured **by** adding lipase enzyme at a level of 0.2 and 0.4%, respectively. All cheese samples were manufactured using 1% active cheese starter culture (1:1), and left until acidity reached the appropriate **level**. To all cheese treatments, 0.02% (w/v) of calcium chloride was added. Rennet was then added (at the level which completed coagulation within 40 min). Then the curd was cut vertically and horizontally with cheese knives and stirred by hand. The temperature was raised gradually to 45 °C within 15 min, the whey was drained off to the level of the curd, and then salt was added at **a** concentration of 2% (w/w) of milk weight and re-stirred for 5 min. The curd was molded and pressed for the first 24 h, **and** pressing was continued overnight after increasing the pressure to enhance the expulsion of whey. Dry salt was added to the surface of the cheese for 5 days while turning it over every day. The cheese molds were coated with cheese wax and stored at about 14 °C for ripening. Three replicates were carried out for each treatment. The resultant Ras cheese was examined when fresh, and after 30, 60, 90, and 120 days for moisture, titratable acidity, pH value, total nitrogen, soluble nitrogen, non-protein nitrogen, tyrosine, tryptophan, total volatile fatty acids, rheological properties, and sensory evaluation.

#### Physico-chemical properties

The moisture, titratable acidity (TA), and ash contents of the Ras cheese were determined according to AOAC [50]. pH values were measured using a digital laboratory pH meter (Jenway 3510, UK). Total nitrogen (TN), soluble nitrogen (SN), and non-protein nitrogen (NPN) of cheese samples were determined by **the** semi-micro **Kjeldahl** distillation method [51]. The fat was determined by using **a** centrifuge at 60 °C for **3–5** minutes at 1300 rpm in a **Gerber Compact centrifuge** (Gerber Instruments AG, Effretikon, Switzerland), and total volatile fatty acids (TVFA) were determined in **the** cheese sample according to the method described by Kosikowski [52]. The soluble tyrosine and tryptophan contents were measured according to Vakaleris and Price [53].

#### Textural profile analysis

Textural profile analysis was performed on the Ras cheese samples according to the method of Glibowski et al. [54] using the double compression test (TA-XT2i texture analyzer, Stable Micro Systems, Godalming, UK). Experiments were carried out by compression tests that generated a plot of force (N) versus time (s). A 25 mm diameter Perspex conical-shaped probe was used to measure the TPA of the Ras cheese samples in their cups, performing five repetitions. In the first stage, the samples were compressed by 30% of their original depth. The speed of the probe was 2 cm/min during the pretest. The following parameters were determined according to the definition given by the International Dairy Federation: Hardness (N) = maximum force of the 1st compression, Cohesiveness = area under the 2nd compression/area under the 1st compression (A2/A1), Springiness (mm) = length of the 2nd compression/length of the 1st compression (L2/L1), Gumminess (N) = Hardness × cohesiveness, and Chewiness (mJ) = gumminess × springiness.

#### Sensory analysis

The sensory analysis was performed according to Davis [55]. The members of the Dairy Science Department, National Research Center, Giza, Egypt, and five consumers as a sample from the Egyptian population (all aged 25–55 years) were invited to participate in the sensory session to evaluate all Ras cheese samples. Cheese samples were organoleptically scored for flavor (50 points), body and texture (40 points), and appearance (10 points) according to the score card.

### Statistical analysis

Statistical analysis was performed according to SAS Institute (1990) [56], using the General Linear Model (GLM) with the main effect of treatments. Duncan’s multiple range test was used to separate the means of the three replicates at *P* ≤ 0.05.

## Results and discussion

### Isolation and quantitative screening of lipase-producing isolates

**Fig. 1:**
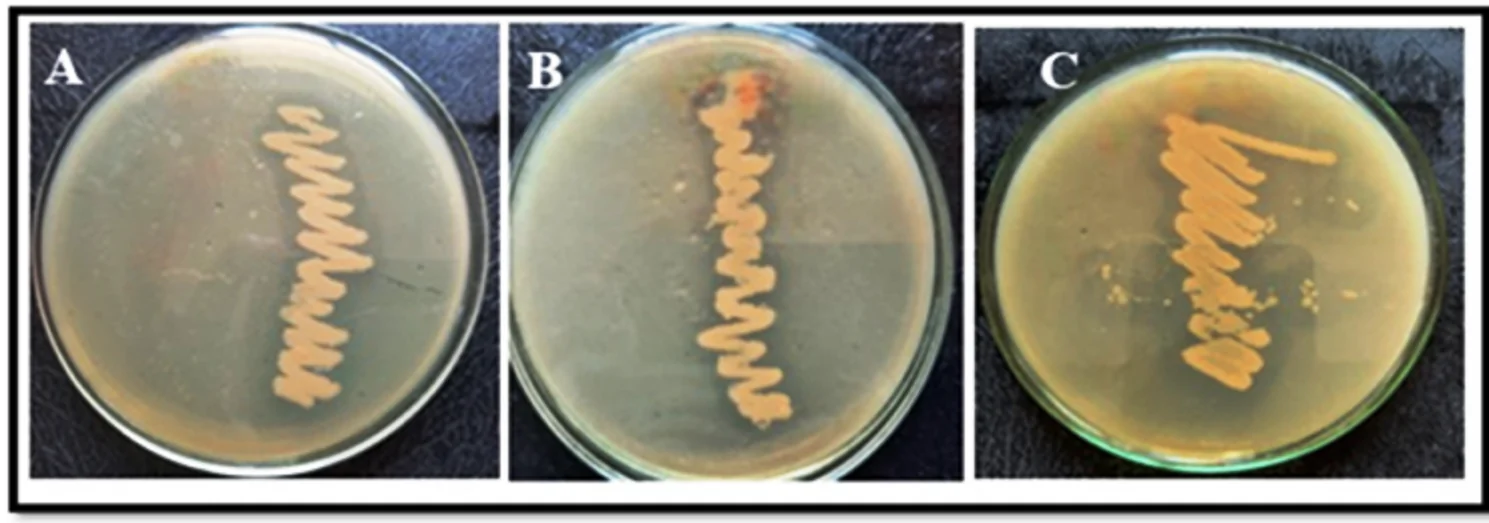
Lipolytic activity of purified bacterial isolates on Tributyrin agar: lipase activity of isolates **A**, **B**, and **C** was determined by the presence of clear zone surrounding bacterial growth on tributyrin agar plates

Three bacterial isolates produced distinct hydrolysis halos on tributyrin agar, providing preliminary qualitative evidence of extracellular lipase/esterase production (Fig. 1). These isolates were designated strains A, B, and C. To quantitatively compare their lipolytic potential, enzyme activity was determined in two production media containing different lipid substrates.

In the tributyrin-containing medium, strain C exhibited the highest lipase activity (6.2 U/mL), followed by strain A (5.2 U/mL), whereas strain B showed the lowest activity (4.1 U/mL). In contrast, when olive oil was used as the sole lipid substrate, only strains C and A exhibited detectable lipase activity. Among them, strain C again showed superior enzyme production, reaching 56.2 U/mL compared with 42.5 U/mL for strain A. Based on its consistently high lipolytic activity in both media, strain C was selected for subsequent molecular identification as well as lipase production, characterization, and application studies. For all subsequent experiments, strain C is referred to as NRC420. The superior lipolytic activity of strain NRC420 in both tributyrin- and olive oil-containing media suggests that this isolate possesses a highly efficient lipase production system with broad substrate specificity. Tributyrin is a short-chain triglyceride that is readily hydrolysed by both esterases and true lipases, making it an effective substrate for the initial screening of lipolytic microorganisms. In contrast, olive oil is composed predominantly of long-chain triglycerides, which are preferential substrates for true lipases. Therefore, the ability of NRC420 to exhibit high activity toward both substrates indicates the production of a true lipase with broad substrate specificity rather than an esterase with limited substrate preference.

Moreover, the consistently high enzyme production in the presence of olive oil suggests that lipid substrates may act as inducers of lipase synthesis in strain NRC420. Lipase production in many bacteria is known to be inducible and is enhanced by hydrophobic substrates such as vegetable oils, which stimulate the expression of lipase-encoding genes and increase extracellular enzyme secretion. The superior performance of NRC420 compared with the other isolates may therefore reflect a greater capacity to sense, utilize, and metabolize lipid substrates, resulting in enhanced lipase biosynthesis. This characteristic is particularly advantageous for industrial applications, where lipases are commonly required to efficiently hydrolyse a wide range of natural triglycerides under different processing conditions. These observations agree with previous reports indicating that tributyrin is an effective substrate for primary screening of lipolytic microorganisms, whereas olive oil serves as a selective inducer for true lipase production [57–59]. Furthermore, several studies have demonstrated that the presence of vegetable oils enhances extracellular lipase production through substrate-induced regulation of lipase gene expression and enzyme secretion [60].

### Molecular identification of the most potent lipase producer and the phylogenetic tree

**Fig. 2:**
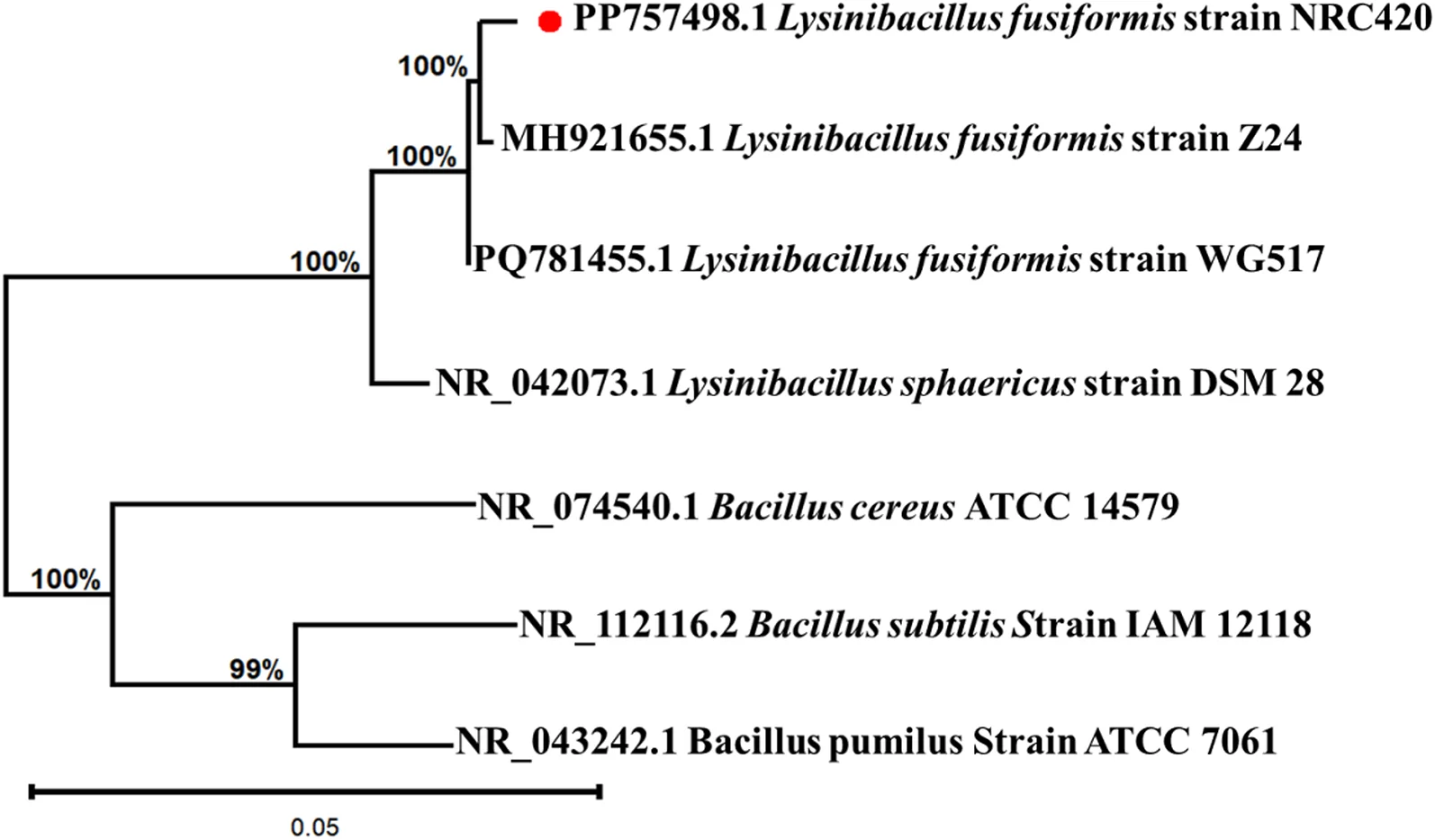
Phylogenetic relationships between bacterial isolate *NRC420* and other relevant bacteria from the GenBank database were analyzed using the neighbor-joining method. Numerical values at nodes indicate bootstrap percentages from 1000 replicates

The 16 S rRNA nucleotide sequence was aligned with related sequences in the GenBank database, revealing 99.49% similarity to *Lysinibacillus fusiformis*. The sequence was submitted to GenBank under accession number PP757498. Phylogenetic analysis using the neighbor-joining method grouped the isolate, designated *Lysinibacillus fusiformis* strain NRC420, with closely related *Lysinibacillus* species (Fig. 2), thereby confirming its taxonomic identity and supporting the BLAST results.

### Molecular study

#### PCR based amplification, cloning of lipase encoding genes, and sequence analysis

**Fig. 3:**
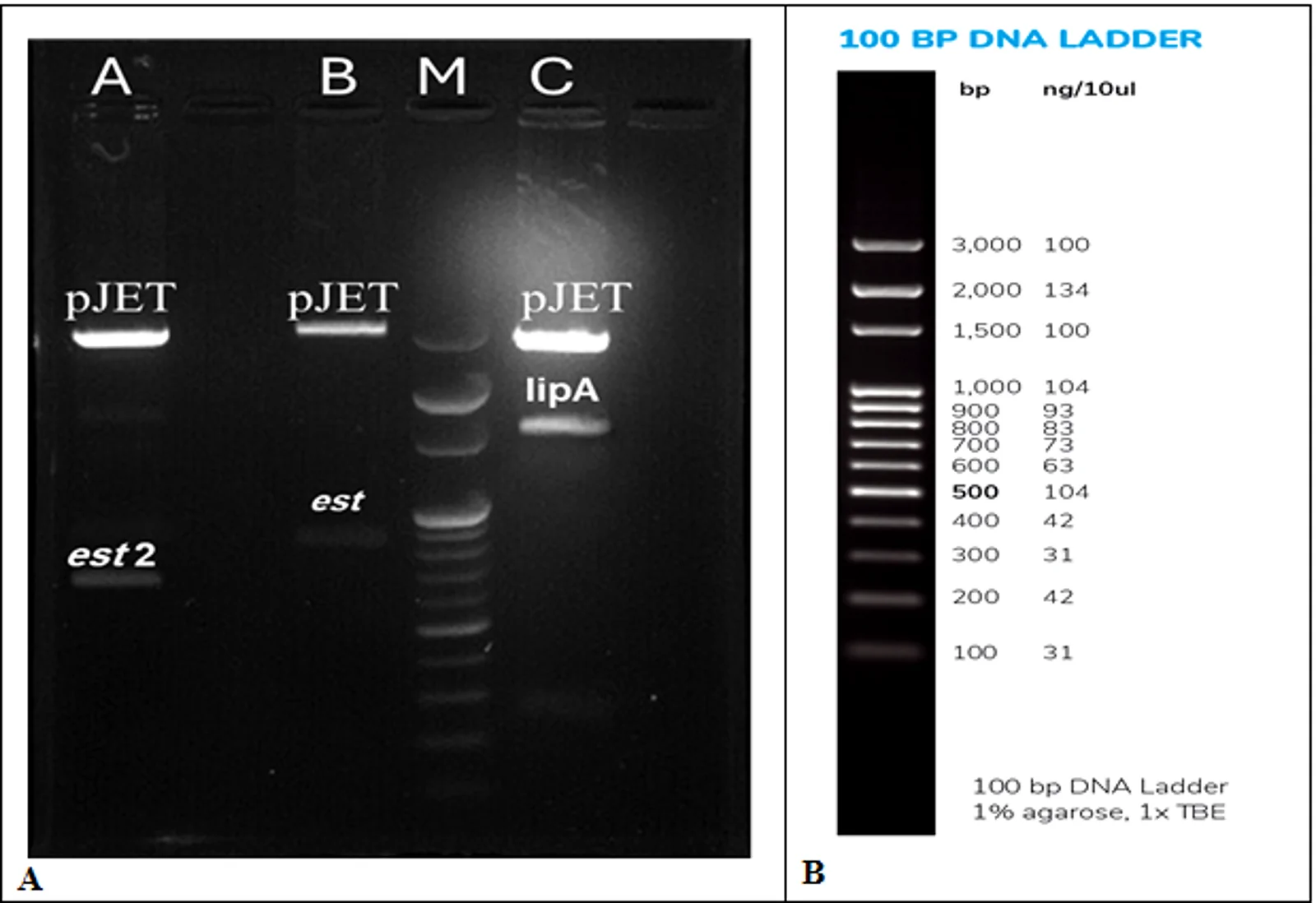
**A**: Validation of est2-pJET (**A**), est-pJET (**B**), and lipA-pJET (**C**) recombinant construct assembly by double restriction digestion, showing the released recombinant gene fragments (indicated by their respective symbols) and the linearized pJET vector. M:100 bp DNA Ladder. **B**: 100 bp DNA Ladder SolisFAST^®^

Prior to this step, each primer pair listed in Table 1 successfully amplified a single DNA fragment corresponding to genes with IDs 29,443,290, 29,439,326, and 29,440,449. The amplified products were 1756 bp, 941 bp, and 687 bp in length, respectively. This preliminary amplification was conducted to verify the specificity of the designed primers. Subsequently, Phusion High-Fidelity DNA Polymerase was employed to generate accurate, proofreading PCR products. Each amplified fragment was individually cloned into the pJET vector. Positive recombinant clones were confirmed by colony PCR and double restriction digestion analysis. Fig. 3 illustrates the double digestion results of the three recombinant vectors, showing the expected insert sizes along with the pJET vector backbone (~ 3000 bp).

**Fig. 4:**
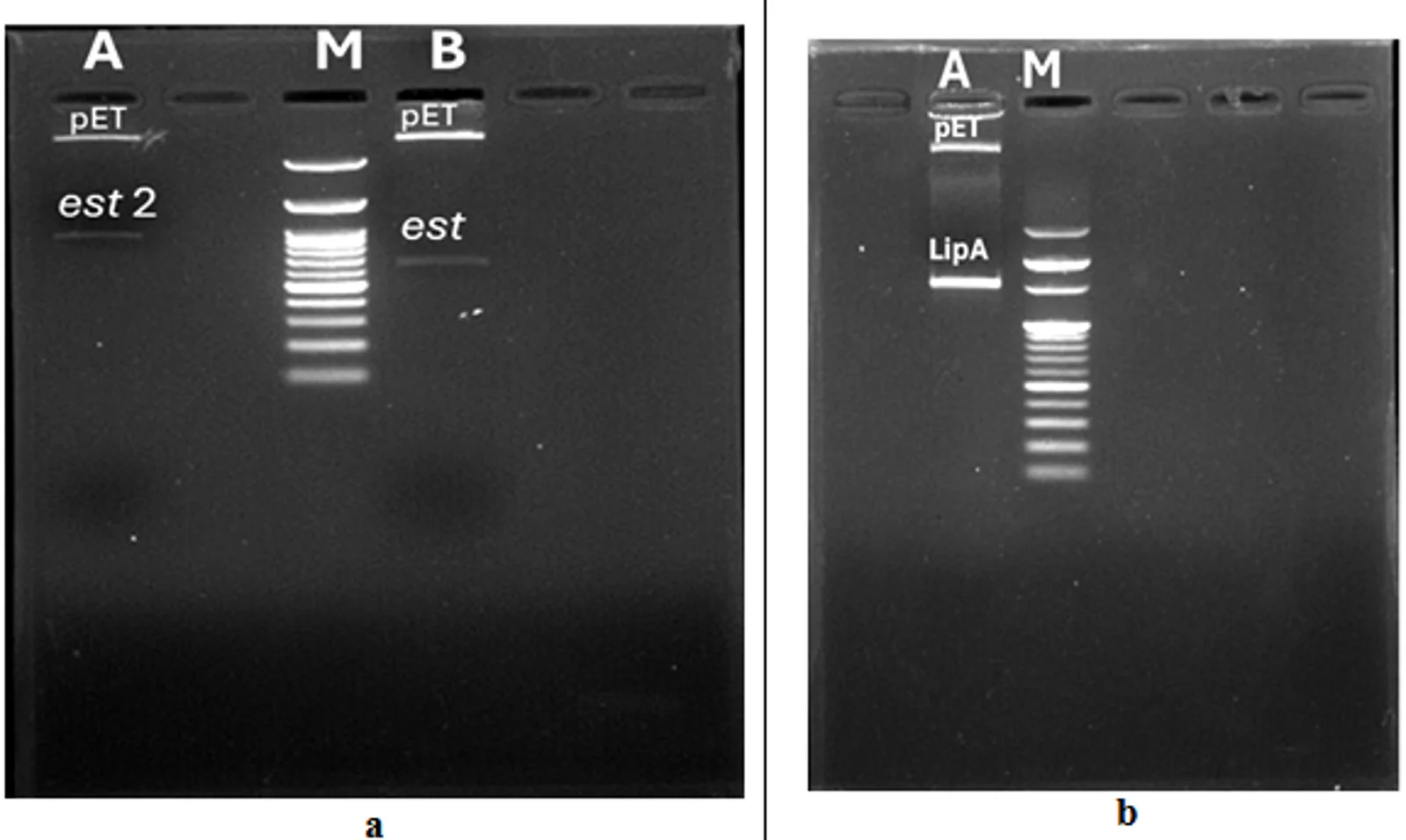
**a**: Validation of **est2-pET** (**A**) and **est-pET (B)** recombinant constructs assembly by double restriction digestion, showing the released recombinant gene fragments (indicated by their respective symbols) and the linearized **pET** vector. **M**: 100 bp DNA ladder H3 RTU (GeneDirex, Inc.) **b**: Validation of **lip-pET** recombinant construct assembly by double restriction digestion (A). **M**: 100 bp DNA Ladder SolisFAST^®^ used for fragment size estimation

The verified recombinant constructs were designated as *est*-pJET, *est2*-pJET, and *lipA*-pJET. These constructs, along with the pET-28a (+) expression vector, were subjected to double digestion using the appropriate restriction enzymes to facilitate subcloning of the target gene fragments into the pET-28a (+) vector. Successful construction of the recombinant expression vectors was confirmed by double restriction digestion, which produced three distinct patterns corresponding to the expected insert sizes along with the pET-28a (+) vector backbone (~ 5369 bp) (Fig. 4). The newly generated expression plasmids were designated as *est*-pET, *est2*-pET, and *lipA*-pET. These constructs were subsequently transformed and propagated in *Escherichia coli* DH5α. Following DNA sequencing and sequence editing, the obtained sequences exhibited 100% identity with the original gene sequences used for primer design. The confirmed sequences were submitted to GenBank and assigned the following accession numbers: PX136937.1 (*est*), PX136938.1 (*est2*), and PX136936.1 (*lipA*).

In this study, two complementary investigations were undertaken. The first focused on the production, purification, and characterization of recombinant lipase from *Lysinibacillus fusiformis*, whereas the second evaluated its potential industrial applications as a detergent additive and in the manufacture of Ras cheese.

During the fermentation of *L. fusiformis* in olive oil-containing medium, lipase production is accompanied by the secretion of other extracellular proteins and constitutively expressed enzymes, which may interfere with enzyme purification and activity determination. Heterologous expression provides an effective strategy to overcome these limitations by enabling the production of a single target enzyme with high purity and yield in a well-characterized host. The first step of recombinant protein production is gene cloning into suitable vector and that can be achieved either by chemical synthesis of the target gene or by PCR amplification of the native gene followed by cloning into a suitable expression vector. In the present study, the latter approach was employed to isolate the lipase-encoding gene from *L. fusiformis* and express it recombinantly for subsequent characterization and application studies. Similar strategies have been successfully applied for bacterial lipases. For example, Ganesh et al. [61] amplified and cloned a lipase-encoding gene for recombinant expression, while He et al. [62] cloned and expressed a cold-active alkaline lipase gene from *Bacillus cereus* U2 in *Escherichia coli*, for subsequent gene expression applications.

Oleate hydratase (rLipase), a flavin adenine dinucleotide (FAD)-dependent enzyme that catalyzes the hydration of unsaturated fatty acids, has been identified in several members of the order *Bacillales*, including the genera *Listeria*, *Lysinibacillus*, *Paenibacillus*, and *Staphylococcus*. A comprehensive sequence similarity network analysis classified 2046 putative Oleate hydratase proteins into 11 homologous families, highlighting substantial sequence diversity, particularly within the catalytic loop region [63, 64]. Sequence analysis of the deduced amino acid sequence in the present study revealed the presence of the highly conserved **GGXXXG** motif, a characteristic signature associated with bacterial lipases and implicated in substrate binding and catalytic activity [65]. In addition, multiple sequence alignment identified the conserved **YGLLSN** serine motif (Figure S1), suggesting the presence of a conserved catalytic region shared among homologous proteins.

#### Heterologous expression of lipase encoding genes and optimization of plasmid-mediated expression

The verified constructs (*est*-pET, *est2*-pET, and *lipA*-pET) were transformed into *Escherichia coli* BL21 (DE3) as the expression host. Enzyme production was monitored under varying IPTG concentrations and incubation temperatures to determine optimal expression conditions. The results demonstrated that both induction temperature and IPTG concentration significantly influenced recombinant lipase expression. The expressed recombinant enzymes were designated as rEst, rEst2, and rLipA. Maximum expression was achieved at 0.2 mM IPTG and an incubation temperature of 18 °C for rEst and rEst2 while 28 °C for rLipA. In this step, the study focused on the production and purification of recombinant lipase enzymes from *Lysinibacillus fusiformis*. Given during bacterial fermentation, multiple extracellular enzymes, including lipases, were produced; however, achieving high-level expression in the native host can be limited by regulatory and metabolic constraints. To overcome these expression bottlenecks, heterologous expression systems are commonly employed. Nevertheless, the efficiency of recombinant protein production strongly depends on the compatibility between the expression vector, host strain, and induction conditions [66]. In the present study, the *Escherichia coli* BL21 (DE3) system combined with the pET28a (+) vector proved to be highly efficient for the production of functional, high-yield recombinant lipase. This expression system has also been successfully applied in previous studies for the production of soluble and active recombinant enzymes [67–69]. For example, El-Sayed et al. [69] used a similar system for the heterologous expression of chitinase B as a potential biocontrol agent against insect pests. Likewise, Karakaş et al. [70] reported successful expression of a cold-active lipase from *Pseudomonas* sp. KE38 in *E. coli*, where the enzyme was partially purified from inclusion bodies and characterized as a 65 kDa protein. In our study, induction conditions were found to be critical for optimizing recombinant protein expression. In particular, IPTG concentration significantly influenced enzyme yield in the pET-28a (+) system. Optimal expression of the recombinant enzymes **rEst**, **rEst2**, and **rLipA** was achieved at 0.2 mM IPTG. Additionally, the incubation temperature played an important role, with 18 °C being optimal for rEst and rEst2, while 28 °C favored maximal expression of rLipA. These conditions collectively enhanced soluble protein production and overall enzymatic yield. Importantly, the presence of additional N-terminal amino acid residues resulting from the expression vector did not negatively affect rLipase activity. This indicates that these residues do not interfere with the enzyme’s folding, active conformation, or catalytic function, thereby preserving its biochemical performance.

### Assessment of recombinant lipase activity and SDS-PAGE electrophoresis

**Fig. 5:**
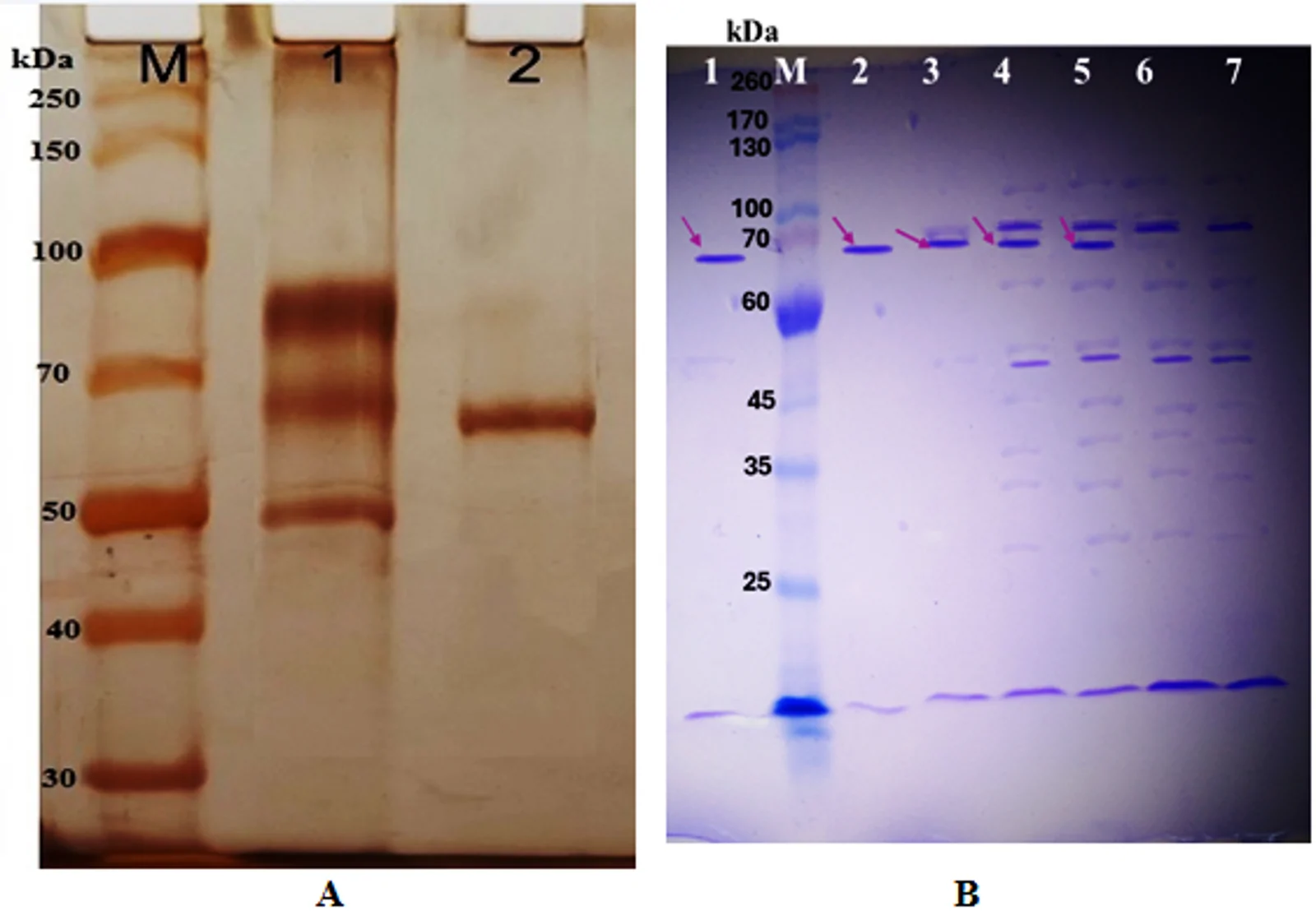
**A** Silver-stained SDS–PAGE of the purified lipase from wild strain showing a single protein band corresponding to the lipase enzyme (lane 2). **B**: Coomassie Brilliant Blue-stained SDS–PAGE showing expression and purification of the recombinant lipase Of *E. coli* BL21 (DE3) cells. **A** Partially purified lipase from the wild strain; lane M: protein ladder; lanes 1 and 2: different protein fractions precipitated with ethanol. **B** Total cellular proteins from *E. coli* BL21 (DE3) cells transformed with the recombinant plasmid pET-LipA. Lane M: protein marker (IRIS11 pre-stained protein ladder, 3–260 kDa). Lanes 6 and 7: uninduced cultures; lanes 4 and 5: IPTG-induced cultures; lanes 1 and 2: partially purified recombinant lipase from IPTG-induced *E. coli* BL21 (DE3). The recombinant lipase band is indicated by pink arrows

Crude extracts from three potential clones carrying the constructs rEst, rEst2, and rLipA were precipitated, and their enzymatic activities were evaluated using the long-chain fatty acid substrate p-nitrophenyl palmitate (PNPP). Among the three recombinant proteins, only rLipA (encoded by the *lipA* gene) exhibited significant lipase activity toward *p*-nitrophenyl palmitate reaching 150 U/mL. In contrast, recEst and recEst2 showed no detectable activity toward this long-chain substrate but exhibited activity toward *p*-nitrophenyl acetate and other short-chain fatty acid esters, indicating that they are esterases rather than true lipases. The molecular weights of the wild-type and recombinant lipase are shown in Fig. 5. The wild-type enzyme exhibited a distinct band at approximately 65 kDa Fig. 5A, whereas the recombinant lipase encoded by rLipA (rLipase) showed a band at approximately 66 kDa. This ~ 1 kDa shift is consistent with the addition of a polyhistidine (His₆)-tag, which typically contributes ~ 0.8–1 kDa to the fusion protein, depending on linker sequences Fig. 5B.

The molecular weights of bacterial lipases vary considerably among species and even among strains, reflecting differences in amino acid sequence, structural organization, and post-translational modifications. Consistent with the present findings, recombinant bacterial lipases have been reported to exhibit molecular masses over a relatively broad range. For example, a recombinant lipase from *Bacillus subtilis* appeared as a single band of approximately 24 kDa following 19.7-fold purification [71]. Similarly, the recombinant lipase from *Bacillus amyloliquefaciens* PS35, heterologously expressed in *Escherichia coli* DH5α, was purified 19.41-fold with a recovery yield of 9.7% and had a molecular weight of approximately 29 kDa [72]. Likewise, the purified recombinant lipase from *Bacillus licheniformis* was reported as a single protein band of approximately 25 kDa [73]. More recently, He et al. [62] reported that the recombinant cold-active alkaline lipase from *Bacillus cereus* U2 had an apparent molecular weight of approximately 38 kDa. Collectively, these studies demonstrate that recombinant bacterial lipases exhibit a wide range of molecular weights (approximately 24–38 kDa), depending on their microbial origin and structural characteristics, and support the successful expression and purification of the recombinant *Lysinibacillus fusiformis* lipase obtained in the present study.

### In silico analysis

#### Assessment of homology modeling

The three-dimensional structure of the deduced amino acid sequence of rLipase was predicted through homology modeling using the MODELLER package. The resulting model was further evaluated using several validation approaches to confirm its structural quality and reliabilityas described by Wiederstein and Sippl [74], Rishad et al. [75], and Oduselu et al. [76]. Verify3D analysis demonstrated acceptable model performance, with 81.86% of residues showing average 3D–1D scores ≥ 0.1. Ramachandran plot analysis (Figure S2) classified residues into four conformational regions: most favored, additionally allowed, generously allowed, and disallowed. Of the total residues, 92.2% were located in the most favored regions, while 7.4% were found in additionally allowed regions, and only 0.4% occurred in generously allowed regions. Collectively, these validation outcomes support the accuracy and structural integrity of the predicted rLipase model.

#### Molecular docking analysis

The interaction between rLipase and the major fatty acids present in olive oil was effectively investigated through computational modeling and molecular docking analysis. Although computational approaches have limitations compared with experimental methods, they remain valuable tools for exploring protein structure–function relationships and substrate–protein interactions in a cost-effective manner [77].

Molecular docking analysis was conducted to evaluate ligand–receptor binding affinities and interaction stability based on key non-covalent interactions, including hydrogen bonding, hydrophobic contacts, and van der Waals forces [78]. Binding affinity was expressed as binding energy (kcal/mol), where more negative values indicate stronger and more stable ligand–receptor interactions [79]. According to established thresholds, binding energies < − 4.25, < −5.0, and < − 7.0 kcal/mol correspond to moderate, good, and strong binding affinities, respectively [80].

**Fig. 6:**
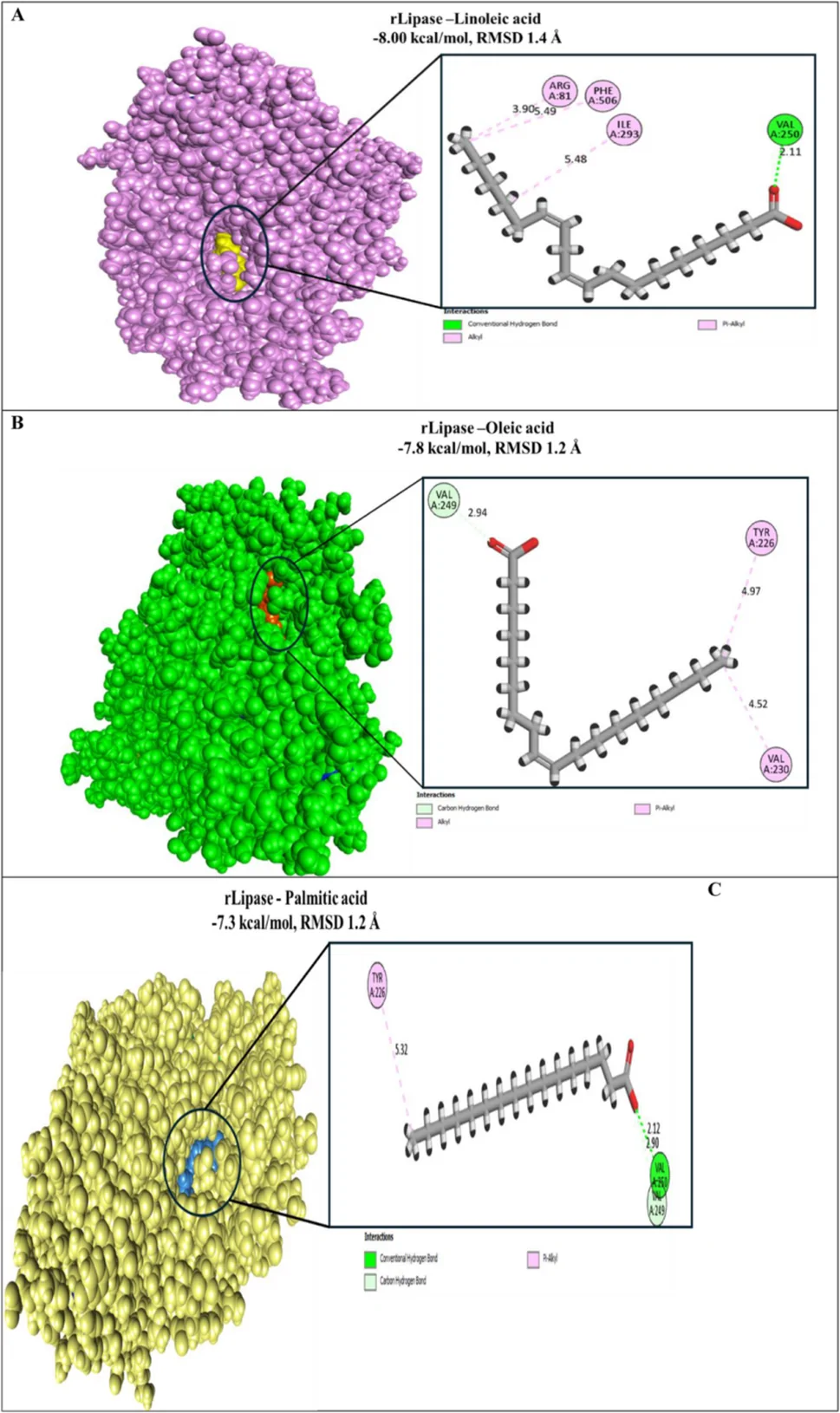
Molecular docking interactions of the rLipase active site with linoleic acid (**A**), oleic acid (**B**), and palmitic acid (**C**), the major fatty acids present in olive oil

As shown in Fig. 6, rLipase exhibited strong binding affinity toward the major fatty acids present in olive oil—oleic acid, linoleic acid, and palmitic acid—with all binding energies falling within the strong affinity range. Among these, linoleic acid showed the strongest interaction with rLipase, with a binding energy of − 8.0 kcal/mol and an RMSD value of 1.4 Å (Fig. 6A), followed by oleic acid (− 7.8 kcal/mol; RMSD 1.2 Å) (Fig. 6B) and palmitic acid (− 7.3 kcal/mol; RMSD 1.2 Å) (Fig. 6C). The high binding stability of the rLipase–linoleic acid complex was supported by a hydrogen bond with VAL250 (2.1 Å), along with hydrophobic interactions involving Alkyl and Pi–Alkyl contacts. Similarly, the interaction with oleic acid was stabilized predominantly through hydrophobic interactions, including Alkyl, carbon–hydrogen bonding, and Pi–Alkyl interactions. In the rLipase–palmitic acid complex, VAL250 played a critical role in hydrogen bond formation (2.1 Å), highlighting its importance in ligand recognition and underscoring its potential role in stabilizing substrate interactions within the rLipase active site. Moreover, additional hydrophobic interactions further contributed to complex stabilization. Collectively, these findings suggest favorable substrate recognition and stable binding of rLipase toward major olive oil fatty acids.

Validation of the docking protocol for the rLipase–fatty acid complexes was supported by two major criteria: binding energy and root-mean-square deviation (RMSD). Strong binding affinities ranging from − 8.0 to − 7.3 kcal/mol, together with low RMSD values of 2.1 Å, support the reliability of the predicted binding poses, as RMSD values below 2.5 Å are generally considered indicative of accurate docking solutions [81]. Overall, these findings demonstrate that in silico analysis is a powerful approach not only for predicting binding efficiency, but also for improving understanding of structural interactions and identifying important amino acid residues associated with catalytic function and activity enhancement [82]. Consistent with this approach, Liu et al. [83] demonstrated that molecular docking effectively revealed the binding mechanism between lipase and lipid substrates during the enzymatic synthesis of diacylglycerol-enriched vegetable oils, providing mechanistic insights into substrate recognition and catalytic efficiency. In support of this, Haryati et al. [81] combined molecular modeling, docking, and molecular dynamics simulations to investigate the interaction of a bacterial lipase with triglyceride substrates, demonstrating that these in silico approaches effectively predict binding stability and enzyme–substrate recognition. Such computational analyses are valuable for understanding lipase catalytic mechanisms and guiding the engineering and selection of enzymes for industrial applications.

### Partial purification of rLipase enzyme

As shown in Table 2, fractional precipitation with chilled ethanol at different saturation levels (25, 50, and 75%, v/v) resulted in the recovery of three lipase-containing fractions. Among them, the 50% ethanol fraction exhibited the highest lipase activity (3885 U/fraction), corresponding to a specific activity of 41.5 U mg⁻¹ protein and a 3.2-fold purification compared with the crude enzyme preparation. Therefore, this fraction was selected for subsequent biochemical characterization and application studies. Partial purification by organic solvent precipitation is a simple, rapid, and economical approach for concentrating extracellular enzymes while simultaneously removing a substantial proportion of contaminating proteins. Ethanol decreases the dielectric constant of the aqueous medium and competes with proteins for water molecules, thereby reducing protein solubility and promoting selective precipitation according to differences in protein hydrophobicity, molecular size, and surface charge. Consequently, proteins possessing different physicochemical properties precipitate at different solvent concentrations, enabling partial purification without the need for sophisticated chromatographic techniques [84, 85].

The superior recovery of rLipase in the 50% ethanol fraction suggests that this solvent concentration provided an optimal balance between enzyme precipitation and structural preservation. Lower ethanol concentrations (25%) were likely insufficient to completely precipitate the enzyme, whereas higher concentrations (75%) may have co-precipitated additional contaminating proteins or partially affected the native conformation of the enzyme, resulting in lower specific activity. Similar observations have been reported for bacterial lipases, where intermediate ethanol concentrations (40–60%) frequently achieve the highest purification efficiency while maintaining catalytic activity [5, 86]. The 3.2-fold increase in specific activity obtained in the present study indicates effective enrichment of the recombinant lipase while preserving substantial catalytic activity. Although this purification fold is modest compared with multi-step chromatographic protocols, it is considered appropriate for industrial biocatalysts, where maximizing enzyme recovery, minimizing processing costs, and maintaining catalytic performance are often more important than achieving electrophoretic purity. For industrial applications such as detergent formulations, food processing, biodiesel production, and biotransformations, partially purified enzymes frequently exhibit performance comparable to highly purified preparations while substantially reducing production costs [87]. Moreover, limiting purification steps minimizes enzyme loss and shortens processing time, thereby improving the overall recovery yield. Recent studies on recombinant microbial lipases have similarly employed solvent precipitation as an effective preliminary purification step before biochemical characterization, demonstrating that partially purified lipases retain excellent catalytic efficiency and operational stability while offering greater economic feasibility for large-scale production [60, 86]. Therefore, the partially purified rLipase obtained at 50% ethanol precipitation represents a suitable preparation for subsequent characterization and industrial application studies.

**Table 2:** Partial purification of recombinant *Lycinibacillus fusiformis* lipase by ethyl alcohol precipitation

Ethyl alcohol concentration (%)	Protein content mg/fraction	Activity of fraction (U)	Specific activity U/mg protein	Recovered protein (%)	Recovered activity (%)	Fold purification
Culture filtrate	435.5	5618	12.9	100	100	1.00
25	36.5	120	3.3	8.4	2.1	0.3
50	93.6	3885	41.5	21.5	69	3.2
75	293.3	145.9	0.5	67.3	2.6	0.04

### Characterization of partially purified *Lycinibacillus fusiformis* rLipase enzyme

#### Effect of reaction temperature

**Fig. 7:**
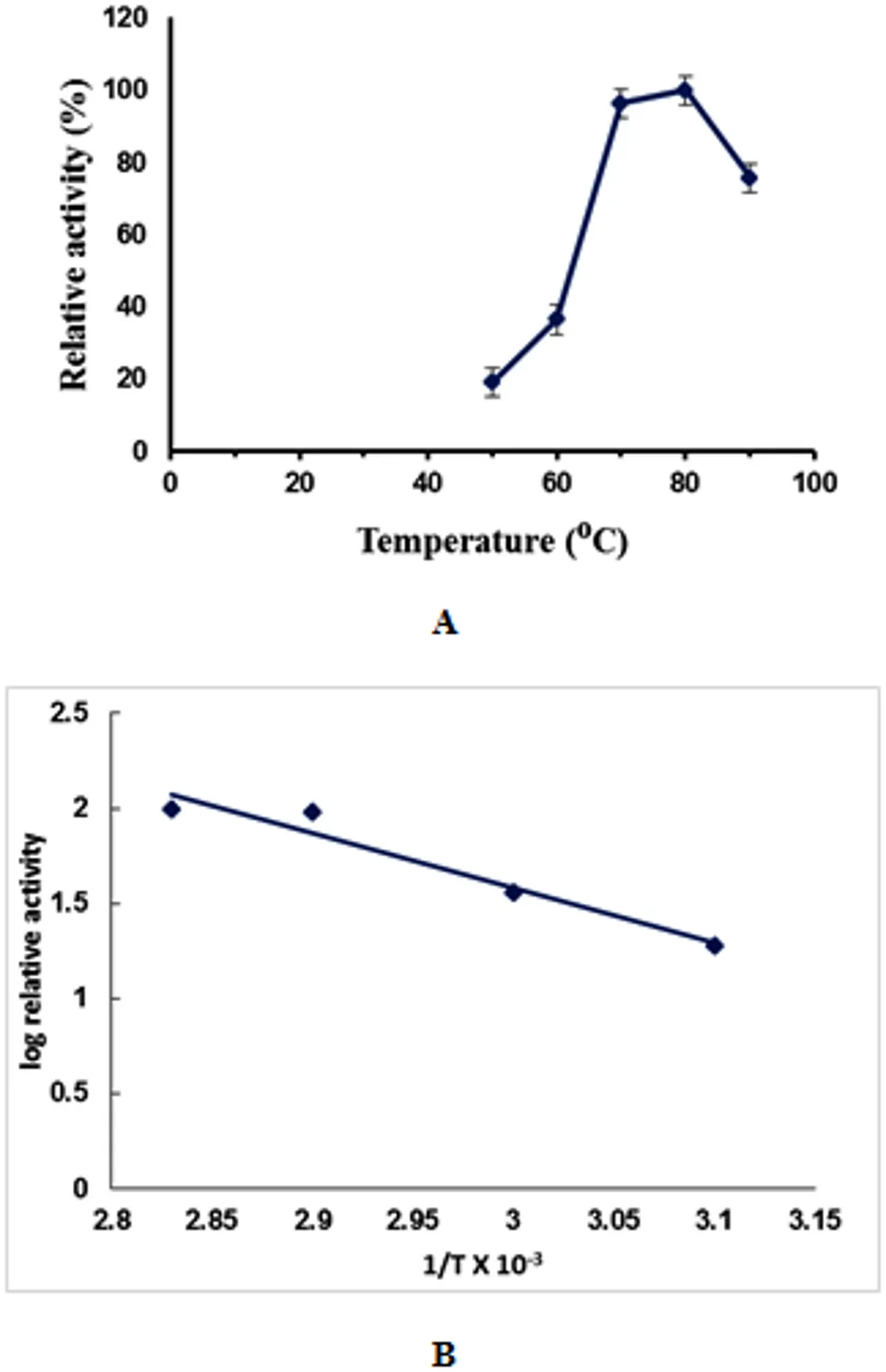
**A** Effect of reaction temperature on *Lycinibacillus fusiformis* rLipase activity, **B**. Arrhenius plot for *Lycinibacillus fusiformis* rLipase enzyme

The partially purified recombinant lipase from *Lysinibacillus fusiformis* exhibited maximum catalytic activity at 80 °C (Fig. 7A), indicating that the enzyme is highly thermotolerant. A marked decline in activity was observed at temperatures above 80 °C, suggesting the onset of thermal denaturation that disrupts the native protein conformation and the integrity of the catalytic site. Temperature is one of the most influential factors affecting lipase activity because it directly impacts substrate diffusion, enzyme flexibility, catalytic turnover, and protein stability. As temperature increases, the kinetic energy of enzyme and substrate molecules also increases, leading to enhanced collision frequency and reaction rates until the optimum temperature is reached. Beyond this point, excessive thermal energy destabilizes non-covalent interactions, including hydrogen bonds, hydrophobic interactions, and electrostatic forces that maintain the enzyme’s three-dimensional structure, resulting in reduced catalytic activity [88].

Compared with previously reported microbial lipases, the recombinant *L. fusiformis* lipase demonstrated a considerably higher optimum temperature. Lipase from *Bacillus subtilis* KM-BS exhibited maximum activity at 55 °C [89], while lipase from *B. subtilis* 168 showed an optimum temperature of 35 °C [90] Therefore, the optimum temperature of 80 °C observed in the present study places this recombinant enzyme among the more thermostable bacterial lipases reported to date. The ability of the present lipase to maintain maximum activity at 80 °C suggests that it possesses structural adaptations that enhance thermal resistance. Thermostable enzymes generally exhibit increased numbers of salt bridges, hydrogen bonds, hydrophobic interactions, compact protein packing, and rigid secondary structural elements, all of which contribute to maintaining the integrity of the catalytic conformation under elevated temperatures [91]. The remarkable thermostability of the recombinant lipase is particularly advantageous for industrial applications. Many industrial processes, including detergent formulations, and food processing.

The activation energy (*Ea*) of the enzymatic reaction, calculated from the Arrhenius plot, was 55.3 kJ mol⁻¹ (Fig. 7B). The activation energy represents the minimum energy required to convert substrate molecules into the transition state during catalysis. The obtained value falls within the range commonly reported for microbial lipases and indicates efficient catalytic performance at elevated temperatures [88].

#### Effect of reaction pH

The optimum activity of partially purified rLipase was observed in alkaline condition at pH 9 (Figure S3). Lower optimum pH values were previously observed for lipase enzyme. For example, the optimum pH of *Penicillium sp.* lipase was observed at pH 4 [92], and for *Lacticaseibacillus rhamnosus* IDCC 3201 lipase, it was reported at pH 8 [93]. On the other hand, for *B. subtilis* 168 lipase, it was reported at pH 10 [90]. This high activity of *Lycinibacillus fusiformis* lipase at alkaline conditions offers an advantage for being applicable in detergent formulation [94]. The activity decreased at pH values lower or higher than the optimum, indicating that the catalytic performance of the enzyme is highly dependent on the pH of the reaction medium. Variations in pH affect the ionization state of amino acid residues involved in substrate binding and catalysis, thereby altering the conformation and catalytic efficiency of the enzyme. Consequently, each lipase exhibits a characteristic pH optimum that reflects its structural adaptation and physiological origin.

In consistence of our findings, the lipase from *Lacticaseibacillus rhamnosus* IDCC 3201 showed maximum activity at pH 8 [93]. In contrast, the lipase from *Bacillus subtilis* 168 exhibited optimum activity at pH 10 [90]. These differences demonstrate the broad diversity of microbial lipases and reflect variations in the amino acid composition surrounding the catalytic triad, as well as differences in enzyme structure and environmental adaptation.

The alkaline optimum (pH 9) observed for the *Lysinibacillus fusiformis* lipase indicates that the enzyme is well suited for applications requiring catalytic activity under basic conditions. Recent studies have highlighted that alkaline bacterial lipases are desirable industrial biocatalysts because they maintain high catalytic efficiency and structural stability in alkaline environments, particularly when combined with elevated temperatures and surfactants. Such properties increase their value in detergent formulation at pH 8–11, where processing is commonly carried out under alkaline conditions [95]. In a similar study by Abol-Fotouh et al. [96], Optimization, purification, and biochemical characterization of thermoalkaliphilic lipase its activity optima 9 from a novel Geobacillus stearothermophilus FMR12 for detergent formulations.

The observed alkaline optimum is particularly advantageous for detergent applications because commercial laundry detergents generally operate at pH 8–11. Under these conditions, lipases hydrolyze triglycerides into glycerol and free fatty acids, facilitating the removal of grease and oil stains from fabrics [97].

#### Effect of some surfactants and commercial detergents on rLipase activity

Surfactants are indispensable components of modern detergent formulations because they reduce surface tension, emulsify hydrophobic stains, and increase the accessibility of lipid substrates to lipolytic enzymes. Consequently, compatibility with surfactants and commercial detergents is one of the primary criteria for selecting lipases intended for detergent applications [11]. The effects of different surfactants and commercial detergents on the activity of *Lysinibacillus fusiformis* rLipase are presented in Table 3. The enzyme exhibited remarkable stability and compatibility in the presence of all tested surfactants. Interestingly, lipase activity was enhanced rather than inhibited, with the highest activity recorded in the presence of SDS. The stimulatory effect of surfactants may be attributed to their ability to increase the oil–water interfacial area, thereby facilitating substrate accessibility and promoting interfacial activation of the lipase. In addition, surfactants can induce subtle conformational changes that maintain the catalytic site in an open and catalytically active state, leading to improved hydrolytic efficiency. Similar stimulation by SDS has been reported for lipases from *Staphylococcus arlettae* [98]. In contrast, SDS inhibited the activity of lipases from *Ralstonia pickettii* [99], highlighting that the response to surfactants is enzyme-specific and largely depends on differences in protein structure, surface hydrophobicity, and the stability of the catalytic domain. The compatibility of *Lysinibacillus fusiformis* lipase with SDS is particularly noteworthy because this anionic surfactant is generally regarded as a strong protein-denaturing agent. The observed tolerance suggests that the enzyme possesses a robust structural architecture capable of maintaining its native conformation and catalytic functionality despite exposure to surfactant molecules. Such stability is a highly desirable characteristic for detergent enzymes, as they are routinely exposed to surfactants during storage and washing processes.

The enzyme also retained high activity in the presence of all tested commercial detergents, confirming its excellent detergent compatibility. The highest activity was obtained with Ariel powder (174 ± 1.4%), followed by Persil (110.6 ± 1.28%), whereas Oxi gel retained comparatively lower activity (93.3 ± 1.06%). The differences observed among detergents are likely attributable to variations in their chemical composition, including the types and concentrations of surfactants, builders, bleaching agents, oxidants, and other additives that may either stabilize or partially inhibit enzyme activity. Compared with the lipase from *Streptomyces* sp. AU-153, which exhibited substantial activity loss in the presence of Persil (93.6 ± 0.54%) and especially Ariel (43.4 ± 2.5%) [11], the *Lysinibacillus fusiformis* lipase demonstrated markedly superior compatibility with commercial detergent formulations. Collectively, these findings demonstrate that rLipase exhibits a combination of biochemical properties that are highly desirable for incorporation into modern laundry detergent formulations, where resistance to alkaline conditions, elevated temperatures, and detergent additives is essential for maintaining efficient lipid stain removal.

**Table 3:** Effect of some surfactants and commercial detergents on rLipase activity

Surfactant (1%)	Lipase relative activity (%)	Detergent (1%)	Lipase relative activity (%)
Control	100	Control	100
SDS	332 ± 0.49	Ariel gel	102.65 ± 3.3
Tween 20	174.25 ± 3.18	Persil gel	110.6 ± 1.28
Tween 80	267 ± 2.8	Oxi gel	93.3 ± 1.06
Triton X-100	286.3 ± 3.25	Ariel powder	174 ± 1.4

#### Effect of some metal ions on rLipase activity

The effects of different metal ions on the activity of the recombinant *Lysinibacillus fusiformis* lipase were evaluated. Calcium ions (Ca²⁺) significantly enhanced lipase activity, resulting in the highest relative activity (123 ± 2.0%), followed by magnesium ions (Mg²⁺), which increased activity to 113 ± 3.04% compared with the control (100 ± 0.2%). In contrast, manganese (Mn²⁺) and copper (Cu²⁺) had only minor effects on enzyme activity, maintaining 93 ± 1.3% and 99 ± 3.18% of the control activity, respectively. Barium ions (Ba²⁺) exhibited the strongest inhibitory effect, reducing the relative activity to 81 ± 1.13%. Similar stimulatory effects of Ca²⁺ on lipase activity have been reported for lipases from other bacterial sources. Wang et al. [100] demonstrated that a novel alkaline lipase from *Acinetobacter calcoaceticus* 1–7 exhibited markedly enhanced catalytic activity in the presence of Ca²⁺, Mg²⁺, and K⁺, whereas Cu²⁺, Al³⁺, Fe³⁺, Ba²⁺, and Zn²⁺ partially inhibited enzyme activity. Likewise, Mg²⁺ has been shown to enhance the activity of the *Bacillus subtilis* lipase [101], indicating that these divalent cations commonly act as positive modulators of bacterial lipases. The stimulatory effect of Ca²⁺ is attributed to its ability to stabilize the lipase structure and catalytic site while reducing product inhibition through the formation of insoluble calcium salts with liberated fatty acids during hydrolysis, thereby enhancing catalytic efficiency [102]. The activation by Mg²⁺ is likely due to its stabilizing effect on the enzyme structure and catalytic center. Although less effective than Ca²⁺, Mg²⁺ has also been reported to enhance the activity of several bacterial lipases [100]. In contrast, the reduction in enzyme activity observed with the other tested metal ions suggests that these ions interfere with catalysis through different mechanisms. Certain metal ions may compete with essential calcium-binding sites or interact with amino acid residues located near the catalytic center, thereby altering the three-dimensional structure of the enzyme and reducing substrate accessibility [96]. Heavy metal ions, in particular, may bind strongly to functional groups such as sulfhydryl, carboxyl, or imidazole residues, leading to conformational changes or partial inactivation of the enzyme. Similar inhibitory effects have been reported for several microbial lipases, where metal ions reduced activity by disrupting the catalytic environment or competing with catalytically favorable ions [103].

### Evaluation of *Lycinibacillus fusiformis* rLipase in detergent formulation

The cleaning efficiency was determined from the difference in the weight of the cotton fabric before and after washing [47]. The recombinant lipase alone achieved a cleaning efficiency of 48%. Furthermore, supplementation of the detergent solution with recombinant rLipase increased the cleaning efficiency from 43.4% to 52%, demonstrating its ability to enhance the removal of oil-based stains. These findings indicate that the recombinant rLipase is a promising detergent additive for improving the removal of lipid stains from cotton fabrics.

This cleaning efficiency of *Lycinibacillus fusiformis* rLipase (48%) was slightly higher than that reported for lipase from *Magnetospirillum gryphiswaldense* (47%) [47]. The improvement in detergent performance observed in the present study (from 43.4 to 52%) was markedly greater than that reported by Gulen et al. [11], who observed only a slight increase in cleaning efficiency from 31.12% to 32.3% following lipase supplementation. The enhanced washing performance is primarily attributed to the enzymatic hydrolysis of triglycerides into glycerol and free fatty acids, which are more readily emulsified and removed by surfactants during washing. The excellent detergent performance of rLipase is further supported by its alkaline activity, high thermostability, and compatibility with surfactants and commercial detergents, all of which are desirable characteristics for modern detergent formulations. In addition, the incorporation of enzymatic additives can reduce the reliance on harsh chemical detergent components, thereby improving cleaning efficiency while minimizing the environmental impact of detergent formulations [104].

### Evaluation of the partially rLipase in accelerating egyptian hard cheese (Ras Type) ripening

#### Physico-chemical properties

As presented in Table 4, the moisture content of fresh Ras cheese ranged from 41.34% to 42.13%, with no significant differences (*p* ≤ 0.05) among the treatments at the beginning of ripening. After 120 days, the moisture content decreased to 35.26–37.02% in all treatments, reflecting the gradual loss of water during ripening. The lower moisture content observed in the control cheese may be attributed to greater moisture loss, whereas cheeses supplemented with recombinant rLipase retained relatively higher moisture throughout the ripening period. Among the enzyme-treated cheeses, T2 exhibited the highest moisture content. The higher moisture retention in the enzyme-treated samples may also be associated with the increased acidity developed during ripening, which influences the water-holding capacity of the cheese matrix. Similar observations have been reported by several authors, who found that enzyme-treated Ras cheese maintained higher moisture than the control throughout ripening, although moisture declined progressively in all treatments as ripening advanced [105–111].

Changes in titratable acidity and pH during ripening are also summarized in Table 5. The addition of recombinant rLipase significantly (*p* ≤ 0.05) increased the acidity of Ras cheese compared with the control throughout the ripening period, accompanied by a corresponding decrease in pH. The increase in acidity is consistent with the biochemical changes that occur during ripening and agrees with previous studies. El-Sayed et al. [112] reported significantly higher acidity in Ras cheese supplemented with Jalapeño red pepper than in the control cheese. Likewise, Abd Allah et al. [110], Hamdy et al. [109], and El Shiekh et al. [105] observed higher acidity in treated Ras cheese than in untreated cheese. Similarly, Mehanna et al. [108] found that the pH of Ras cheese decreased significantly during the first 30 days of ripening and remained relatively stable thereafter.

The total nitrogen (TN) content of the control and rLipase-treated Ras cheeses is presented in Table 5. TN gradually increased in all treatments throughout ripening, reaching 4.99–5.10% after 120 days. This increase is primarily attributed to the progressive reduction in moisture content, which concentrates the protein fraction. At the end of ripening, the enzyme-treated cheeses showed significantly (*p* ≤ 0.05) higher TN values than the control. These findings agree with Mehanna et al. [108], who reported greater lipolysis in enzyme-treated cheeses than in the control. Similarly, several studies have demonstrated a significant increase (*p* < 0.05) in TN during ripening, together with marked differences among treatments, mainly as a consequence of moisture loss and the concentration of cheese solids [113, 114].

In addition to changes in moisture and protein content, the concentrations of ash, salt, and fat increased significantly (*p* ≤ 0.05) in the rLipase-treated cheeses compared with the control during ripening. These increases are largely attributable to the reduction in moisture content, which results in the concentration of cheese constituents. In contrast, Ivanova et al. [115] reported no significant (*p* > 0.05) changes in dry matter, total protein, fat, or salt contents during the ripening of cheese over a 60-day period, indicating that compositional changes may vary depending on cheese type, ripening conditions, and the use of enzyme supplementation.

**Table 4:** The chemical composition of Ras cheese made by different concentrations of lipase enzyme during ripening period

Parameter	Storage period (Days)	Treatments		
		Control	T1	T2
Moisture (%)	Fresh	41.34^a^_±0.08_	41.38^a^_±0.62_	42.13^a^_±0.02_
	30	39.55^b^_±0.27_	39.31^b^_±0.27_	39.16^b^_±0.15_
	60	38.54^b^_±0.82_	38.06^c^_±0.22_	38.47^c^_±0.21_
	90	36.97^c^_±0.56_	37.07^d^_±0.56_	37.56^d^_±0.24_
	120	35.26^d^_±0.49_	36.83^e^_±0.77_	37.02^d^_±0.31_
SE		0.36	0.29	0.18
pH	Fresh	5.86^a^_±0.03_	5.82^a^_±0.01_	5.78^a^_±0.01_
	30	5.41^b^_±0.04_	5.30^b^_±0.55_	5.41^b^_±0.05_
	60	4.86^c^_±0.04_	5.12^c^_±0.02_	4.74 ^c^_±0.02_
	90	4.32^d^_±0.10_	4.61^d^_±0.03_	4.25^d^_±0.03_
	120	4.20^d^_±0.08_	4.23^c^_±0.01_	4.01^c^_±0.01_
SE		0.069	0.030	0.023
Titratable acidity (%)	Fresh	0.87^e^_±0.01_	0.81^d^_±0.01_	0.82^d^_±0.02_
	30	1.72^d^_±0.04_	1.49^c^_±0.10_	1.74^c^_±0.09_
	60	1.99 ^c^_±0.15_	1.97^b^_±0.20_	2.02^b^_±0.11_
	90	2.04^ab^_±0.13_	2.08^a^_±0.02_	2.11^a^_±0.02_
	120	2.09^a^_±0.07_	2.10^a^_±0.05_	2.17^a^_±0.06_
SE		0.027	0.020	0.025
TN (%)	Fresh	3.85^c^_±0.07_	3.93^d^_±0.01_	3.94^d^_±0.13_
	30	4.33 ^b^_±0.08_	4.72^c^_±0.03_	4.59^c^_±0.03_
	60	4.82 ^a^_±0.03_	4.92^b^_±0.12_	4.82^b^_±0.08_
	90	4.95^a^_±0.03_	5.04^a^_±0.13_	4.94^b^_±0.11_
	120	4.99^a^_±0.05_	5.10^a^_±0.04_	5.04^a^_±0.04_
SE		0.054	0.024	0.016
Fat %	Fresh	25.83^e^_±0.12_	25.95^e^_±0.08_	26.55^e^_±0.03_
	30	27.15^d^_±0.22_	28.88^d^_±0.19_	28.57^d^_±0.09_
	60	29.90^c^_±0.02_	30.75^c^_±0.04_	31.66^c^_±0.11_
	90	31.17^b^_±0.08_	32.86^b^_±0.05_	32.65^b^_±0.09_
	120	32.32^a^_±0.04_	33.98^a^_±0.12_	33.94^a^_±0.13_
SE		0.013	0.027	0.016
Ash (%)	Fresh	4.49^d^_±0.02_	4.82^d^_±0.03_	4.72^d^_±0.03_
	30	4.51^c^_±0.03_	4.83^c^_±0.04_	4.79^c^_±0.04_
	60	4.65^b^_±0.03_	4.89^b^_±0.03_	4.84^b^_±0.03_
	90	4.69^a^_±0.03_	5.11^a^_±0.07_	5.01^a^_±0.07_
	120	4.71^a^_±0.2_	5.16^a^_±0.02_	5.09^a^_±0.02_
SE		0.016	0.025	0.016
Salts	Fresh	3.11^d^_±0.02_	3.15^d^_±0.01_	3.17^c^_±0.07_
	30	3.58^c^_±0.12_	3.49^c^_±0.12_	3.91^c^_±0.12_
	60	3.86^bc^_±0.02_	3.94^b^_±0.21_	4.08^b^_±0.17_
	90	4.19^b^_±0.14_	4.29^a^_±0.09_	4.17^b^_±0.06_
	120	4.24^a^_±0.08_	4.31^a^_±0.06_	4.28^a^_±0.08_
SE		0.030	0.010	0.014

Means sharing the same letter are not significantly different. Control: traditional Ras cheese prepared without enzyme. T1 and T2: treatments with the addition of 0.2% and 0.4% lipase enzyme, respectively. SE: standard error

#### Ras cheese ripening indices

**Fig. 8:**
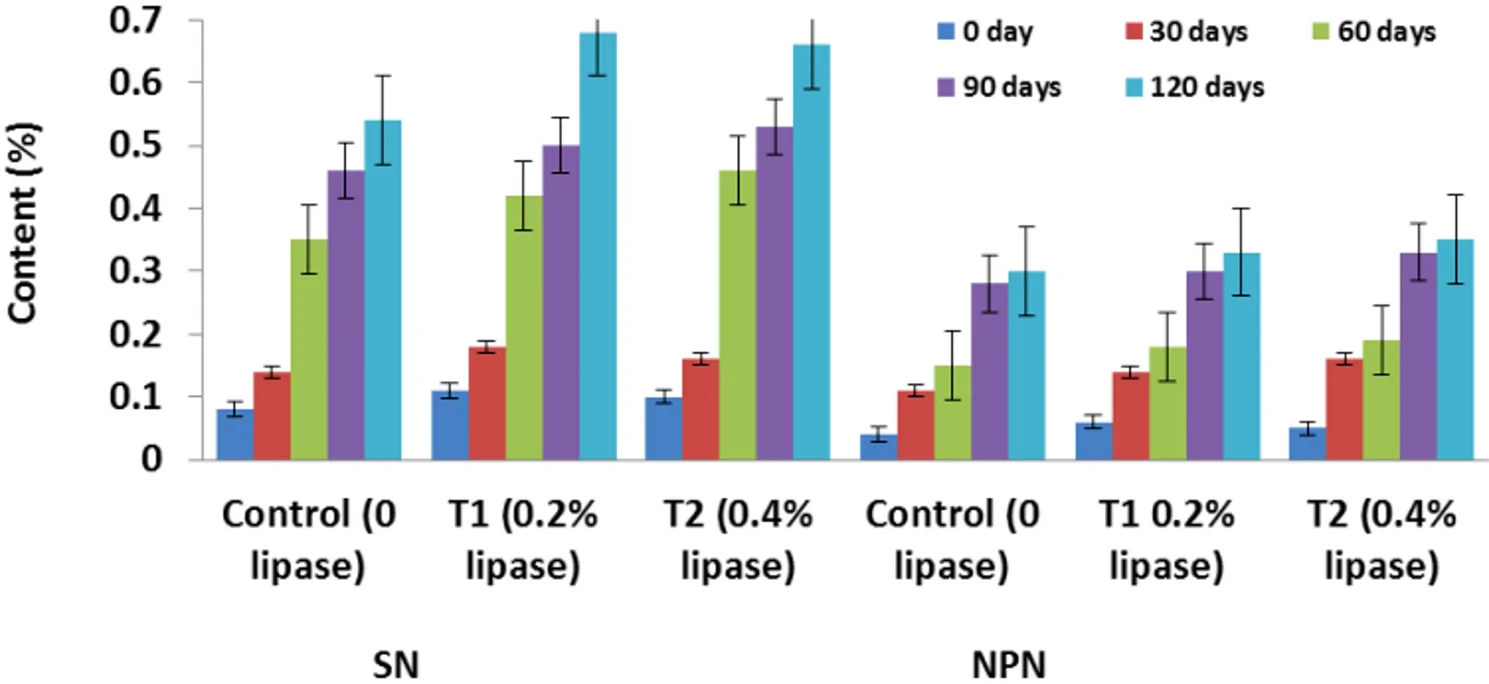
The soluble nitrogen (SN) and Non-protein nitrogen (NPN) contents in the experimental RAS cheese samples during ripening period.

Figures 8 illustrates the changes in soluble nitrogen (SN) and non-protein nitrogen (NPN) contents of the experimental Ras cheese during the ripening period. Both SN and NPN increased progressively in all treatments as ripening advanced, reflecting the continuous proteolytic changes occurring during cheese maturation. At the beginning of ripening, no significant differences (*p* > 0.05) were observed among the treatments for either SN or NPN. However, as ripening progressed, significant differences (*p* ≤ 0.05) became evident. Cheeses supplemented with 0.2% (T1) and 0.4% (T2) recombinant rLipase exhibited the highest SN contents, whereas T2 showed the highest NPN content, differing significantly (*p* ≤ 0.05) from the other treatments.

The progressive increase in SN and NPN is an indicator of proteolysis during ripening and is mainly attributed to the action of proteinases and peptidases produced by the starter culture. The higher SN and NPN values observed in the rLipase-treated cheeses throughout ripening suggest that recombinant rLipase indirectly promoted proteolysis, possibly by enhancing lipolysis and generating conditions that facilitate proteolytic enzyme activity. Consequently, the addition of recombinant rLipase accelerated the formation of soluble peptides and free amino compounds compared with the control cheese. Similar findings have been reported by several authors, who observed progressive increases in SN and NPN during Ras cheese ripening and attributed these changes to the combined activities of starter culture enzymes and ripening-associated proteolysis [108, 109]. Likewise, Khalifa and Wahdan [116] and Abbas et al. [117] reported significant increases in both SN/TN and NPN/TN ratios with increasing ripening time, confirming that these indices are reliable indicators of cheese ripening and protein degradation.

##### Soluble tyrosine and tryptophan (mg/100 g cheese)

Figures S4 shows the changes in soluble tyrosine and tryptophan contents of Ras cheese during the ripening period. Both amino acids increased progressively in all treatments, reflecting the continuous proteolysis that occurred during cheese maturation. At the beginning of ripening, the soluble tyrosine contents were 20.65, 19.98, and 20.65 mg/100 g cheese in the control, T1, and T2 treatments, respectively. After 120 days of ripening, these values increased markedly to 102.45, 127.12, and 133.45 mg/100 g cheese, respectively. The rLipase-treated cheeses exhibited significantly higher (*p* ≤ 0.05) soluble tyrosine contents than the control at the end of ripening, indicating more extensive protein degradation in the enzyme-treated cheeses. A similar trend was observed for soluble tryptophan, another important indicator of cheese ripening. The soluble tryptophan content increased gradually throughout the ripening period in all treatments, rising from 19.51, 20.42, and 21.33 mg/100 g cheese at day 0 to 79.79, 84.79, and 91.46 mg/100 g cheese after 120 days in the control, T1, and T2 treatments, respectively. Among the treatments, T2 (0.4% recombinant rLipase) exhibited the highest soluble tryptophan content and differed significantly (*p* ≤ 0.05) from the other treatments at the end of ripening.

The higher concentrations of soluble tyrosine and tryptophan in the rLipase-treated cheeses indicate that recombinant rLipase accelerated the ripening process by promoting more extensive proteolysis. Although lipases primarily hydrolyze milk triglycerides, lipolysis and proteolysis are closely interconnected during cheese ripening. The free fatty acids released during lipolysis contribute to flavor development and may alter the cheese matrix, facilitating the action of proteinases and peptidases on caseins. Consequently, enhanced lipolysis is often accompanied by increased production of peptides and free amino acids, resulting in higher soluble tyrosine and tryptophan contents [118, 119].

Tyrosine and tryptophan are widely accepted biochemical indicators of cheese ripening because they accumulate as caseins are progressively hydrolyzed into peptides and free amino acids by residual coagulant, starter culture proteinases, non-starter lactic acid bacteria, and indigenous milk enzymes [20]. Therefore, their continuous increase throughout ripening reflects the advancement of proteolysis and is closely associated with the development of cheese flavor and texture. Similar increases in soluble tyrosine and tryptophan during Ras cheese ripening have been reported by Mehanna et al. [108], El Shiekh et al. [105], Hamdy et al. [109], and Abd Allah et al. [110]. The higher values observed in the rLipase-treated cheeses in the present study demonstrate that rLipase accelerated cheese ripening by enhancing the biochemical changes responsible for cheese maturation.

##### Total volatile fatty acids (TVFA)

**Fig. 9:**
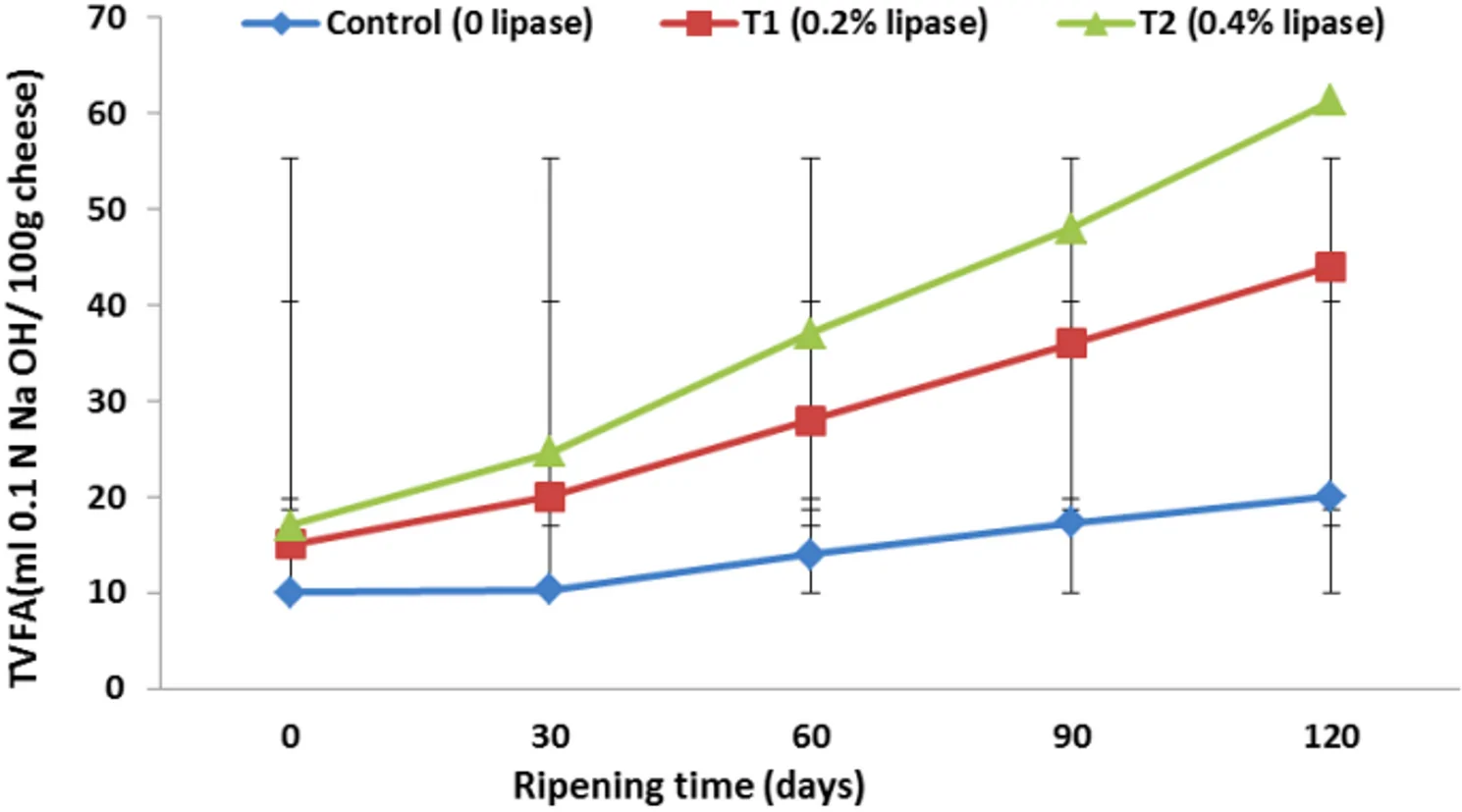
The total volatile fatty acids (TVFA) in Ras cheese treatments. Data are presented as mean ± SD (*n* = 3). Error bars represent standard deviation

As shown in Fig. 9, the total volatile fatty acid (TVFA) content of Ras cheese supplemented with different concentrations of recombinant lipase (rLipase) was significantly (*p* < 0.05) higher than that of the control throughout the ripening period. Moreover, TVFA levels increased progressively in all treatments during ripening, with the highest values observed in the rLipase-treated cheeses at the end of the storage period. The increase in TVFA can be primarily attributed to the enhanced lipolytic activity of the added rLipase, which accelerated the hydrolysis of milk triglycerides into free fatty acids. Short- and medium-chain free fatty acids are highly volatile and constitute the major fraction of TVFA. Besides contributing directly to the characteristic sharp and piquant flavor of ripened cheeses, these compounds serve as important precursors for numerous volatile aroma compounds, including methyl ketones, secondary alcohols, esters, lactones, and aldehydes that develop during cheese maturation [120, 121] Therefore, the higher TVFA values observed in the enzyme-treated cheeses indicate enhanced lipolysis and more advanced flavor development compared with the control. The gradual increase in TVFA throughout ripening is consistent with the continuous action of both the supplemented recombinant lipase and the indigenous lipolytic enzymes derived from milk and starter microorganisms. As ripening progresses, the extent of triglyceride hydrolysis increases, leading to the accumulation of volatile fatty acids and flavor compounds [118]. Similar findings were reported by El-Sayed et al. [112], who observed significantly higher TVFA values in lipase-treated Ras cheese than in the control throughout the ripening period. Comparable increases in TVFA have also been reported in other cheese varieties supplemented with microbial lipases, confirming that exogenous lipase accelerates lipolysis and enhances flavor development by increasing the release of volatile fatty acids [121].

Overall, the present results demonstrate that supplementation with recombinant lipase effectively accelerated lipolysis during Ras cheese ripening, resulting in higher TVFA production. This enhanced formation of volatile fatty acids is expected to promote the development of the characteristic flavor and aroma of ripened Ras cheese while shortening the time required to achieve desirable sensory attributes.

#### Textural profile analysis (TPA)

The textural profile analysis (TPA) results of Ras cheese are presented in Table 5. The texture parameters were significantly influenced (*P* ≤ 0.05) by both the concentration of recombinant lipase (rLipase) and the ripening period. Among the evaluated parameters, hardness, gumminess, and chewiness exhibited noticeable changes throughout storage. The control cheese exhibited significantly higher hardness than the rLipase-treated cheeses throughout the ripening period, while T2 (0.4% rLipase) showed the lowest hardness values, followed by T1. The lower hardness observed in the enzyme-treated cheeses suggests that rLipase promoted a softer cheese matrix. This effect can be attributed to enhanced lipolysis, which modifies the structural integrity of the fat globules and weakens fat–protein interactions within the cheese matrix. In addition, the higher extent of proteolysis observed in the treated cheeses, as evidenced by the increased soluble nitrogen and soluble tyrosine contents, may have further contributed to the softening of the protein network. Similar reductions in cheese hardness following lipase supplementation have been reported by Abd El-Salam et al. [114].

Regardless of treatment, hardness increased gradually and significantly (*P* ≤ 0.05) during the 120-day ripening period. This increase is primarily associated with the progressive loss of moisture, resulting in a more concentrated protein matrix and increased total solids, both of which contribute to firmer cheese texture. Furthermore, the gradual decline in pH during ripening promotes protein aggregation and contraction of the casein network, thereby increasing cheese firmness. Similar increases in hardness during cheese maturation have been reported by several authors [122, 123], who attributed these changes mainly to moisture loss and structural rearrangement of the protein matrix during ripening. Gumminess was also significantly affected by both rLipase concentration and ripening time. Although differences among treatments were relatively small at the beginning of storage, they became more pronounced as ripening progressed. At the end of storage, T2 exhibited the lowest gumminess values, whereas the control cheese showed the highest values. The lower gumminess of the enzyme-treated cheeses is likely related to their reduced hardness, since gumminess is directly influenced by hardness and the integrity of the cheese matrix. Nevertheless, gumminess increased progressively in all treatments throughout ripening, reflecting the gradual development of a denser and less hydrated cheese structure. A similar trend was observed for chewiness, which increased significantly in all treatments during the 120-day ripening period. However, the control cheese consistently exhibited higher chewiness than the rLipase-treated cheeses, whereas T2 showed the lowest values. Because chewiness is closely associated with hardness, cohesiveness, and springiness, the lower chewiness of the treated cheeses reflects the softening effect of enhanced lipolysis and proteolysis on the cheese matrix. As ripening proceeds, moisture loss and matrix compaction increase the energy required to masticate the cheese, explaining the overall increase in chewiness during storage [124].

Overall, the results demonstrate that supplementation with recombinant lipase modified the texture of Ras cheese by producing a softer and less gummy cheese while maintaining the normal textural changes associated with ripening. The reduction in hardness, gumminess, and chewiness in the rLipase-treated cheeses is consistent with the enhanced lipolysis and proteolysis observed during maturation, indicating that recombinant lipase can accelerate biochemical changes that contribute to improved texture and faster cheese ripening.

**Table 5:** Textural profile analysis of Ras cheese fortified by rLipase enzyme during ripening period

Textural properties	Ripening Period (day)	Treatments		
		Control	T1	T2
Hardness N	0	8.15^d^_±0.08_	7.80^d^_±0.01_	7.13^d^_±0.08_
	45	20.42^c^_±0.03_	11.90^c^_±0.18_	13.43^c^_±0.05_
	90	22.11^b^_±0.01_	18.71^b^_±0.01_	20.12^b^_±0.04_
	120	30.93^a^_±0.07_	27.80^a^_±0.02_	24.90^a^_±0.01_
SE		0.012	0.041	0.052
Cohesiveness (B/A area)	0	0.284^d^_±0.02_	0.459^c^_±0.11_	0.439^c^_±0.18_
	45	0.347^c^_±0.01_	0.683^a^_±0.01_	0.613^a^_±0.04_
	90	0.481^b^_±0.06_	0.466^c^_±0.04_	0.412^c^_±0.05_
	120	0.736^a^_±0.09_	0.584^b^_±0.08_	0.564^b^_±0.06_
SE		0.13	0.21	0.18
Springiness Mm	0	0.735^d^_±0.18_	0.741^d^_±0.04_	0.722^d^_±0.01_
	45	0.988^b^_±0.45_	0.897^c^_±0.01_	0.863^c^_±0.11_
	90	0.878^c^_±0.05_	0.952^b^_±0.08_	0.921^b^_±0.03_
	120	1.773^a^_±0.05_	1.352^a^_±0.04_	1.231^a^_±0.05_
SE		0.074	0.071	0.082
Gumminess N	0	2.314^d^_±0.03_	3.580^c^_±0.12_	3.130^c^_±0.18_
	45	7.085^c^_±0.2_	8.127^b^_±0.11_	8.232^b^_±0.38_
	90	10.63^b^_±0.02_	8.718^b^_±0.05_	8.289^b^_±0.28_
	120	22.76^a^_±0.07_	16.235^a^_±050_	14.04^a^_±0.17_
SE		0.044	0.042	0.048
Chewiness N/mm	0	1.701^d^_±0.07_	2.652^d^_±0.08_	2.382^d^_±0.07_
	45	6.999^c^_±0.02_	7.289^c^_±0.07_	7.104^c^_±0.05_
	90	9.333^b^_±0.05_	8.299^b^_±0.03_	7.634^b^_±0.06_
	120	40.35^a^_±0.08_	21.95^a^_±0.02_	17.28^a^_±0.01_
SE		0.055	0.074	0.055

Means sharing the same letters are not significantly different. C: control (traditional Ras cheese prepared without enzyme). T1 and T2: treatments with the addition of 0.2% and 0.4% lipase enzyme, respectively. SE: standard error. N: Newton; mm: millimeters; N/mm: Newton/mm

#### Sensory evaluation of Ras cheese

Sensory properties are among the most important quality attributes determining consumer acceptance of dairy products. These characteristics include appearance, body and texture, flavor, and the overall acceptability of the product. The sensory evaluation scores of Ras cheese during ripening are presented in Table 6. Regarding appearance, T2 cheese (0.4% rLipase) received the highest scores at the beginning of storage and throughout the ripening period, followed by T1 and the control. In general, the appearance scores improved as ripening progressed, reflecting the gradual development of the characteristic color and surface characteristics of Ras cheese. However, the appearance score of T2 showed a slight decline after 60 days, whereas the control and T1 treatments continued to improve until the end of ripening. This reduction may be associated with the accelerated biochemical changes induced by the higher rLipase concentration, which may have slightly affected the visual characteristics of the cheese at the later stages of maturation.

Body and texture scores also improved during ripening in all treatments, indicating the progressive development of the desired cheese consistency. At the end of the 120-day ripening period, T1 (0.2% rLipase) achieved the highest body and texture score (36.09), followed by T2 (35.29) and the control (35.12). Although T2 exhibited superior texture during the early stages of ripening, its sensory score gradually declined after 60 days, while T1 continued to improve until 90 days before showing a slight decrease toward the end of ripening. In contrast, the control cheese showed a gradual improvement throughout the entire storage period. These findings suggest that the addition of rLipase accelerated cheese ripening by promoting lipolysis and, indirectly, proteolysis, leading to earlier development of desirable textural properties. However, excessive lipolysis at the higher enzyme concentration may have resulted in slight softening of the cheese matrix, which reduced panelists’ preference during the later stages of ripening. Similar observations have been reported for enzyme-accelerated cheese ripening, where moderate lipase supplementation improved texture, whereas excessive lipolysis adversely affected body and texture characteristics [118, 120].

Flavor scores increased progressively in all treatments during ripening (Table 6). At the beginning of storage, T2 exhibited the highest flavor score (41.68), whereas the control received the lowest score (40.11). After 90 days, T2 remained the most preferred treatment (44.38), followed by T1 (43.38) and the control. Interestingly, T1 continued to improve in flavor until the end of ripening, reaching a score of 44.14 after 120 days. These results indicate that supplementation with rLipase enhanced flavor development and accelerated the attainment of desirable sensory characteristics compared with the control cheese.

The superior flavor acceptance of the rLipase-treated cheeses can be attributed to the enhanced lipolysis promoted by the recombinant enzyme, which increased the release of free fatty acids that directly contribute to cheese flavor and serve as precursors for numerous volatile aroma compounds, including methyl ketones, esters, alcohols, and lactones [120, 121]. Moreover, the higher soluble nitrogen content observed in the treated cheeses reflects enhanced proteolysis, generating peptides and free amino acids that also contribute to flavor formation through secondary catabolic reactions [121]. The combined effects of lipolysis and proteolysis therefore explain the higher sensory scores of the enzyme-treated cheeses. Overall, the results suggest that supplementation with recombinant lipase accelerated flavor and texture development, with 0.2% rLipase providing the most balanced sensory quality after 120 days of ripening.

**Table 6:** Sensory evaluation of Ras cheese fortified by rLipase enzyme during ripening period

Characteristics	Storage period (days)	Treatments		
		Control	T1	T2
Appearance (10 points)	Fresh	6.82^c^_±0.03_	7.33^c^_±0.02_	7.41^c^_±0.01_
	30	7.11^b^_±0.01_	7.82^b^_±0.07_	7.90^b^_±0.02_
	60	7.63^b^_±0.03_	8.73^ab^_±0.98_	8.50^a^_±0.08_
	90	8.18^a^_±0.07_	8.85^a^_±0.05_	8.20^a^_±0.09_
	120	8.30^a^_±0.02_	8.95^a^_±0.02_	7.31^d^_±0.06_
SE		0.15	0.084	0.21
Body and texture (40 points)	Fresh	32.25^d^_±0.09_	35.54^c^_±0.08_	36.44^c^_±0.05_
	30	32.39^d^_±011_	35.66^c^_±0.11_	36.76^b^_±0.03_
	60	33.68^c^_±0.03_	36.28^b^_±0.13_	37.38^a^_±0.08_
	90	34.62^b^_±0.09_	36.70^a^_±0.07_	36.73^b^_±0.09_
	120	35.12^a^_±0.04_	36.09^ab^_±012_	35.29^d^_±0.04_
SE		0.48	0.51	0.27
Flavor (50 points)	Fresh	40.11^d^_±0.11_	40.68^e^_±0.02_	41.68^d^_±0.05_
	30	41.16^c^_±0.02_	41.11^d^_±0.06_	42.11^c^_±0.03_
	60	42.27^b^_±0.08_	42.05^c^_±0.12_	43.05^b^_±0.07_
	90	42.49^b^_±0.13_	43.38^b^_±0.09_	44.38^a^_±0.11_
	120	43.81^a^_±0.09_	44.14^a^_±0.08_	43.14^b^_±0.09_
SE		0.35	0.39	0.31
Total Score (100)	Fresh	79.18^e^_±0.11_	83.55^e^_±0.032_	85.53^e^_±0.056_
	30	80.66^d^_±0.057_	84.59^d^_±0.22_	86.77^d^_±0.24_
	60	83.58^c^_±0.12_	87.06^c^_±0.14_	88.93^c^_±0.088_
	90	85.29^b^_±0.095_	88.93^b^_±0.089_	89.31^b^_±0.067_
	120	87.23^a^_±0.075_	89.18^a^_±0.055_	85.74^a^_±0.079_
SE		0.089	0.076	0.12

Means sharing the same letters are not significantly different. C: control (traditional Ras cheese prepared without enzyme). T1 and T2: treatments with the addition of 0.2% and 0.4% lipase enzyme, respectively. SE: standard error

The overall sensory acceptability of the Ras cheese samples is presented in Table 6. After 120 days of ripening, T1 (0.2% rLipase) achieved the highest overall acceptability score (89.18), followed by the control (87.23) and T2 (0.4% rLipase) (85.74). These results indicate that supplementation with 0.2% rLipase produced the most acceptable cheese at the end of the ripening period (*P* < 0.05). In contrast, T2 reached its highest overall acceptability score (89.31) after only 90 days of ripening, which was comparable to the score attained by T1 after 120 days. This earlier achievement of maximum sensory quality suggests that the higher concentration of rLipase accelerated the ripening process by enhancing both lipolysis and proteolysis, as reflected by the higher soluble nitrogen and volatile fatty acid contents observed in the treated cheeses. These biochemical changes generate peptides, free amino acids, and free fatty acids that act as precursors for the formation of characteristic cheese flavor compounds [118–121].

Since the primary objective of this study was to accelerate Ras cheese ripening, the results demonstrate that supplementation with 0.4% rLipase effectively reduced the time required to obtain highly acceptable sensory characteristics from 120 to approximately 90 days. The earlier development of desirable flavor and texture reflects the accelerated degradation of milk proteins and fat, indicating a faster ripening rate compared with the control cheese. Similar observations were reported by Mehanna et al. [108], who found that Ras cheese manufactured with cheese slurry exhibited significantly higher sensory scores than the control due to accelerated ripening.

Overall, the findings demonstrate that recombinant lipase can be successfully used to accelerate Ras cheese ripening while maintaining desirable sensory quality. Although 0.2% rLipase produced the highest overall acceptability after the full ripening period, the 0.4% concentration achieved comparable sensory quality approximately one month earlier. Therefore, supplementation with 0.4% rLipase represents a promising approach for shortening the ripening period, thereby reducing production costs and improving manufacturing efficiency without compromising cheese quality.

## Conclusion

This study demonstrated the successful production and application of a recombinant lipase from *Lysinibacillus fusiformis*. Heterologous expression in *Escherichia coli* BL21 (DE3) using the pET28a(+) expression system significantly enhanced enzyme production, yielding approximately 2.7-fold higher lipase activity than the native enzyme. The partially purified rLipase exhibited excellent activity at extreme thermal and alkaline pH conditions, together with good compatibility with detergent components, making it suitable for application in detergent formulation. The enzyme showed promising performance as a detergent additive by improving the oil stain removal efficiency of the detergent. In addition, its application in Ras cheese manufacture accelerated cheese ripening through enhanced lipolysis and proteolysis, resulting in higher levels of ripening indices, improved flavor development, and desirable modifications in cheese texture. Notably, supplementation of the starter culture with 0.4% rLipase had shortened the cheese ripening period from 120 to 90 days which help in reducing the production costs with maintaining the cheese quality. Overall, rLipase from *Lysinibacillus fusiformis* represents a robust and multifunctional biocatalyst with considerable potential for both detergent and dairy applications. Its ability to enhance cleaning performance and accelerate cheese ripening highlights its industrial relevance and provides a practical approach to improve process efficiency and reduce production costs.

## Supplementary Information

Below is the link to the electronic supplementary material.


Supplementary Material 1.


## Data Availability

No datasets were generated or analysed during the current study.
