# Supplementary material for: Recombinant thermotolerant alkaline lipase from Lysinibacillus fusiformis for detergent and hard (Ras) cheese applications: cloning, expression, molecular docking, and characterization
Source: Microb Cell Fact. 2026 Aug 29;25:178. doi: 10.1186/s12934-026-03084-w (PMC13526305; doi:10.1186/s12934-026-03084-w)
Supplement: Supplementary file 1 [file 12934_2026_3084_MOESM1_ESM.docx]

**Figure S1**. Structure-based multiple sequence alignment showing the evolutionary relatedness and homology degree among Oleate hydratases: WP_354471757.1:1-590 oleate hydratase of *Lysinibacillus parviboronicapiens*, PX136936: Lysinibacillus fusiformis strain NRC420 oleate hydratase (lipA), WP_010767499: oleate hydratase of *Enterococcus phoeniculicola*, WP_071131647.1: oleate hydratase of *Enterococcus timonensis*, WP_161902384: oleate hydratase of *Candidatus Enterococcus willemsii*, WP_414842266: oleate hydratase of *Enterococcus saccharolyticus*, WP_202059328: oleate hydratase of *Enterococcus sp.* BWR-S5, WP_067393559.1:1-590 oleate hydratase of *Enterococcus canis*, WP_161900597: MULTISPECIES: oleate hydratase of Enterococcus, WP_049527134: oleate hydratase of Streptococcus, WP_262996349: oleate hydratase of *Macrococcus capreoli*, WP_111597976: oleate hydratase of *Chitinophaga skermanii*, WP_138188883: oleate hydratase of *Paenibacillus alvei*. The Conserved catalytic motif **GGXXXG** is marked with blue stars and outlined by blue box., while **the conserved YGLLSN serine motif** **is marked with red stars and outlined by red box**

**Figure S2.** PROCHECK plot and statistics analysis of rLipase receptor model

|  | Relative activity % |
| --- | --- |
| 7.5 | 62.8333 |
| 8 | 68.3 |
| 8.5 | 98.9 |
| 9 | 100 |
| 9.5 | 85.266 |
| 10 | 63.93 |
| 10.5 | 32.93 |

**Figure S3.** Effect of reaction pH on recombinant *Lycinibacillus fusiformis* lipase activity.

Data are presented as mean ± SD (n = 3). Error bars represent standard deviation

mg / 100 g cheese

| Soluble tyrosine Soluble tryptophan | 0 day | 30 days | 60 days | 90 days | 120 days |
| --- | --- | --- | --- | --- | --- |
| Control (0 lipase) | 20.65 | 33.16 | 51.74 | 75.07 | 102.45 |
| T1 (0.2% lipase) | 19.98 | 39.5 | 58.41 | 63.74 | 127.12 |
| T2 (0.4% lipase) | 20.65 | 43.5 | 65.07 | 73.74 | 133.45 |
| Control (0 lipase) | 19.51 | 29.22 | 48.76 | 59.69 | 79.79 |
| T1 (0.2% lipase) | 20.42 | 35.89 | 51.09 | 65.03 | 84.79 |
| T2 (0.4% lipase) | 21.33 | 39.56 | 58.42 | 70.03 | 91.46 |

**Figure S4.** The soluble tyrosine and the soluble tryptophan contents in Ras cheeses**.**

Data are presented as mean ± SD (n = 3). Error bars represent standard deviation
